# Penetration of Nanobody-Dextran Polymer Conjugates through Tumor Spheroids

**DOI:** 10.3390/pharmaceutics15102374

**Published:** 2023-09-22

**Authors:** Peter Bitsch, Eva S. Baum, Irati Beltrán Hernández, Sebastian Bitsch, Jakob Harwood, Sabrina Oliveira, Harald Kolmar

**Affiliations:** 1 Institute for Organic Chemistry and Biochemistry, Technical University of Darmstadt, Peter-Grünberg-Str. 4, 64287 Darmstadt, Germany; peter.bitsch@tu-darmstadt.de (P.B.); sebastian.bitsch@tu-darmstadt.de (S.B.); jakob.harwood@stud.tu-darmstadt.de (J.H.); 2 Cell Biology, Neurobiology and Biophysics, Department of Biology, Faculty of Science, Utrecht University, Padualaan 8, 3584 CH Utrecht, The Netherlands; e.s.baum@uu.nl (E.S.B.); i.beltranhernandez@uu.nl (I.B.H.); 3 Pharmaceutics, Department of Pharmaceutical Sciences, Faculty of Science, Utrecht University, Universiteitsweg 99, 3584 CG Utrecht, The Netherlands; 4 Centre of Synthetic Biology, Technical University of Darmstadt, 64287 Darmstadt, Germany

**Keywords:** nanobodies, dextran, photosensitizer, tumor spheroid penetration, photodynamic therapy

## Abstract

Here we report the generation of nanobody dextran polymer conjugates (dextraknobs) that are loaded with small molecules, i.e., fluorophores or photosensitizers, for potential applications in cancer diagnostics and therapy. To this end, the molecules are conjugated to the dextran polymer which is coupled to the C-terminus of an EGFR-specific nanobody using chemoenzymatic approaches. A monovalent EGFR-targeted nanobody and biparatopic version modified with different dextran average molecular weights (1000, 5000, and 10,000) were probed for their ability to penetrate tumor spheroids. For monovalent Cy5-labeled dextraknobs, the utilization of smaller sized dextran (MW 5000 vs. 10,000) was found to be beneficial for more homogeneous penetration into A431 tumor spheroids over time. For the biparatopic dual nanobody comprising MW 1000, 5000, and 10,000 dextran labeled with photosensitizer IRDye700DX, penetration behavior was comparable to that of a direct nanobody-photosensitizer conjugate lacking a dextran scaffold. Additionally, dextraknobs labeled with IRDye700DX incubated with cells in 2D and 3D showed potent cell killing upon illumination, thus inducing photodynamic therapy (PDT). In line with previous results, monovalent nanobody conjugates displayed deeper and more homogenous penetration through spheroids than the bivalent conjugates. Importantly, the smaller size dextrans did not affect the distribution of the conjugates, thus encouraging further development of dextraknobs.

## 1. Introduction

Cancer ranks as the leading cause of death and was responsible for an estimated 10 million deaths worldwide in 2020. Consequently, the development and improvement of new and existing strategies in treatment are of constant interest in research [1,2,3,4,5]. Among these, the concept of antibody–drug conjugates (ADCs) has proven to be a highly promising approach with currently 13 successfully clinically approved ADCs [6]. Hereby, a monoclonal antibody (mAb) serves as a targeting molecule to which a molecular linker is conjugated, linking the mAb to a cytotoxic cargo. Thus, the high target specificity of the mAb, in combination with the potency of the drug, results in improved treatment efficacy and reduction of off-target effects known from unspecific chemotherapy [4,7,8,9,10].

The attachment of payload to the mAb could be achieved by a variety of conjugation methods [9,10,11,12,13]. However, the cytotoxic payloads usually exhibit rather hydrophobic behavior, thus increasing the overall hydrophobicity of the conjugate and thereby limiting the maximum ratio of cargo per targeting molecule [14]. Moreover, a direct correlation has been found between construct hydrophobicity and adverse pharmacokinetic properties [15]. Even though, for example, the reduction of mAb disulfide bonds and subsequent introduction of the drug via Michael-addition could yield a rather homogenous product with a drug to antibody ratio (DAR) up to 8, constructs with high DARs tend to aggregate and precipitate due to insufficient hydrophilicity [14,16,17,18]. Additionally, conjugation strategies aiming for high DAR rely on the merely unselective attachment of payload via chemical conjugation strategies yielding heterogeneous products, even though addressing cysteines of the hinge region of an mAb results in a relatively low heterogeneity of final conjugates [18,19,20].

To address these issues, the concept of dextramabs was introduced by Schneider et al. [21]. Here, to overcome the issue of self-aggregation of high DAR ADCs, trastuzumab was equipped with the polysaccharide dextran serving as a linker scaffold which was decorated with the highly hydrophobic cytotoxin MMAE (monomethyl auristatin E) [21]. Thus, the benefits of both aspects, high drug payload and retained biodistribution properties due to the hydrophilicity of the attached polysaccharide, were combined [21]. DARs of up to 11 were reached by multiple attachment of MMAE molecules to the dextran scaffold, while hydrophilicity of the ADC was retained and even increased in comparison to the unmodified parental antibody [21]. Recently, Tian et al. reported the generation of dextran–doxorubicin prodrug nanoparticles conjugated with CD147 monoclonal antibodies, that, upon acid-sensitive disassembly in the tumor environment and endocytosis, could efficiently inhibit tumor growth [22], further reinforcing the potential of dextran in drug design.

Although these and other conjugation approaches and linkers have been explored to improve ADCs further [23], full-length mAbs and nanoparticle conjugates are known to exhibit poor tissue penetration ability, which limits the targeting efficacy when deep and homogeneous penetration through the tumor is mandatory. Additionally, a comparably long blood clearance of mAbs hinders applications like fluorescence imaging where the high blood half-life of fluorescently marked mAbs leads to reduced contrast at early time points post-administration [24]. Thus, antibody fragments such as a single-chain Fv fragment (scFv, 30 kDa) or a binding domain derived from camelid heavy chain antibodies, named nanobody (NB, 15 kDa), have been already successfully employed as alternative targeting proteins [9,10,25]. As documented by us [26,27,28,29] and others [30,31], conjugates utilizing smaller targeting molecules were shown to penetrate faster and more homogenously into malignant tissue than conjugates of mAbs.

Nevertheless, to equip these smaller targeting molecules with a cargo of desire is challenging, since most published conjugates do not exhibit degrees of conjugation (DOCs) higher than 1 [10]. This might be attributed to the lack of selective conjugation methods capable of multiple attachment of cargos. Even enzyme recognition tags for bioconjugation still limit the number of possible cargos to one [32,33,34]. On the other hand, hydrophobic cargos have a higher impact on overall hydrophilicity of the construct, therefore further limiting the DOC [10,26]. This suggests that construct design still seems to be a trade-off between high payload and good penetration properties.

**Figure 1:**
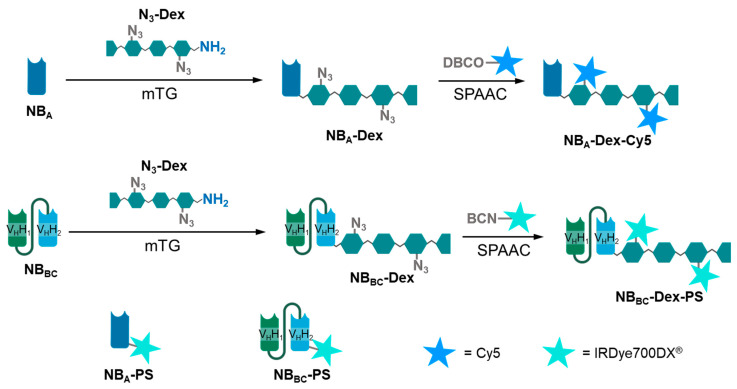
Design of NB_A_-dextran conjugates labeled with Cy5 and NB_BC_-dextran conjugates labeled with IRDye700DX^®^. NB_A_ and NB_BC_ were also directly labeled with IRDye700DX^®^. The number of stars per conjugate should be taken as reference only. Further details on the DOC are provided in Table 1. Created with Biorender.

In this study, we chose to examine whether the previously described concept of dextramabs could be applied to nanobodies. Therefore, nanobody–dextran conjugates (dextraknobs) were generated and decorated with a cargo of desire. Anti-EGFR NBs NB_A_ [35] and the biparatopic 7D12-9G8 [36] (in the following denoted as NB_BC_) were engineered to allow enzyme-mediated conjugation with dextran scaffolds of different molecular weight at the C-terminus (Figure 1). After conjugation, the cargo of desire was introduced via strain-promoted azide–alkyne cycloaddition (SPAAC). For imaging studies, to assess the effect of the dextran on the distribution capacity, we introduced a Cy5 label to the dextraknobs and examined the penetration behavior in 3D tumor spheroids. To assess the potency of this approach in therapeutic applications, we chose the photosensitizer (PS) IRDye700DX^®^ as pro-drug. PSs are a class of pro-drugs that, upon irradiation with light of a certain wavelength, generate reactive oxygen species, causing severe damage to cells, and leading to cell death through different mechanisms [37]. Due to the local light activation, they feature a comparably low to no “dark toxicity” in the absence of light. We have shown the great potency and cytotoxic specificity of nanobody–photosensitizer conjugates [26,27,28,38], and here we have investigated the same PS in NB-dextran-PS conjugates, through EGFR-targeted photodynamic therapy (PDT) in 2D and 3D studies.

## 2. Materials and Methods

### 2.1. Solvents and Reagents

Solvents were obtained from Thermo Fisher Scientific (Waltham, MA, USA) and Carl Roth GmbH & Co. KG (Karlsruhe, Germany) with the following quality: diethyl ether, dichlormethane (DCM), dimethyl sulfoxide (DMSO), methanol (MeOH): synthesis grade; acetonitrile (MeCN): HPLC grade. For HPLC, Millipore quality water was used. All solvents were used without further purification or drying.

Reagents were obtained from Sigma-Aldrich (Darmstadt, Germany), abcr GmbH (Karlsruhe, Germany), Fisher Scientific, Acros Organics (Taufkirchen, Germany), Carl Roth GmbH & Co. KG, and Iris Biotech GmbH (Marktredwitz, Germany). Resin was supplied by Agilent Technologies (Santa Clara, CA, USA). Reagents were used without further purification or drying.

### 2.2. Protein Production and Purification

Expression and purification of NB_A_ and NB_BC_ variants equipped with a Myc-His tag at the *C*-terminus, that were utilized for direct conjugation, were performed as described previously [27].

Expression of NB_A_ variant for enzymatic conjugation was conducted by the inoculation of 50 mL of dYT-media containing 50 µg/mL kanamycin with *E. coli* BL21 DE3 transformed with pET30 expression plasmid containing H6-NB_A_-SPI7G with pelB leader sequence. Cells were incubated overnight at 37 °C and 180 rpm. Thereafter, 1 L of dYT or SB media containing 50 µg/mL kanamycin was inoculated by the overnight culture; OD600 was set to 0.1 and cells were incubated at 37 °C. After OD600 reached 0.8–1.0, IPTG was added to a final concentration of 1 mM and the temperature was reduced to 25 °C. Production was conducted overnight, which was followed by cell harvest the next morning via centrifugation at 4000× *g*.

Expression of NB_BC_ variant for enzymatic conjugation was conducted by inoculation of 50 mL of dYT-media containing 50 µg/mL kanamycin and chloramphenicol with *E. coli* T7ShuffleXpress transformed with pET30 expression plasmid containing H6-NB_BC_-SPI7G. Cells were incubated overnight at 37 °C and 180 rpm in dYT medium. Thereafter, 1 L of dYT medium containing 50 µg/mL kanamycin and chloramphenicol was inoculated by the overnight culture, OD600 was set to 0.1 and cells were incubated at 37 °C. After OD600 reached 0.6, IPTG was added to a final concentration of 1 mM and the temperature was reduced to 25 °C. Production was conducted overnight, which was followed by cell harvest the next morning via centrifugation at 4000× *g*.

Microbial transglutaminase was produced essentially as described by proenzyme expression in *E. coli* with pET30 expression plasmid containing H6-(Pro)-mTG, followed by enzyme activation using neutral protease (Dispase, Worthington). Activated mTG was purified by cation chromatography as described [39].

For nanobody purification, *E. coli* cells from 1 l bacterial culture were resuspended in 20 mL of IMAC A buffer (20 mM imidazole, 20 mM Tris, 300 mM NaCl, pH 7.2) after centrifugation. Cells were lysed by ultrasonication if intracellular products were expected. For periplasmatic expressed products, cells were frozen in suspension overnight at −80 °C and thawed again. In both methods, cells were then centrifugated at 15,000× *g* and the supernatant was filtrated by a 0.45 µM syringe filter.

For His-tagged variants, His-Tag IMAC was performed utilizing an ÄKTA Start (GE Healthcare Life Science, Uppsala, Sweden) equipped with a HisTrap HP purification column (GE Healthcare Life Science). IMAC A served as binding and washing buffer; IMAC B (250 mM imidazole, 20 mM Tris, 300 mM NaCl, pH 7.2) was used for elution. After elution, proteins were transferred into a dialysis tube with a molecular weight cut off of 6000–8000 and incubated overnight at 4 °C in 5 L of 1× PBS.

### 2.3. Analytical Section

#### 2.3.1. High-Performance Liquid Chromatography

High-performance liquid chromatography (HPLC) was performed as reversed-phase (RP) chromatography utilizing an Agilent LC 1100 series HPLC device with diode array detector (DAD). Absorption was measured at 220, 280 nm and 554 nm. An Agilent Eclipse Plus C18 (100 × 4.6 mm, 3.5 m, 95 Å) column was used and eluents consisted of eluent A (0.1% (*v*/*v*) TFA in LC-MS-grade water) and eluent B (0.1% (*v*/*v*) TFA in 90% LC-MS-grade MeCN/water). Three minutes of isocratic flow (starting concentration eluent B) was followed by 20 min gradient flow. After that, the column was washed with 100% eluent B for four minutes and subsequent isocratic flow with starting concentration of eluent B for five minutes.

For hydrophobic interaction chromatography (HIC), an Agilent 1260 infinity series device with variable wavelength detector (VWD) was used with a TSKGel Butyl-NRP (4.6 × 35 mm, 2.5 µM) column (Tosoh Bioscience, Griesheim, Germany). Absorption was measured at 220 nm. Eluents consisted of Eluent A (1.5 M (NH_4_)_2_SO_4_, 25 mM Tris pH 7.5) and Eluent B (25 mM Tris pH 7.5). Two and a half minutes of isocratic flow (0% eluent B) was followed by a 25 min gradient flow to reach 100% B. After that, the column was washed with 100% eluent B for two and a half minutes and subsequent isocratic flow 0% B for five minutes.

#### 2.3.2. Liquid Chromatography–Mass Spectrometry (LC-MS)

For LC-MS analysis, a Shimadzu LCMS-2020 mass spectrometer (Kyoto, Japan) equipped with a Phenomenex Synergy 4 μ Fusion-RP 80 (C-18, 250 × 4.6 mm, 2 m, 80 Å) column (Phenomenex, Torrance, CA, USA) was used. Eluents consisted of eluent A (0.1% (*v*/*v*) formic acid in LC-MS-grade water) and eluent B (0.1% (*v*/*v*) formic acid in 90% LC-MS-grade MeCN/water). The measured unit was the extinction (E) at 220 and 280 nm.

#### 2.3.3. Photometric Measurements

Photometric extinction measurements were carried out on a UVmini-1240 Photometer from Shimadzu (Kyoto, Japan) with a 10 mm quartz SUPRASIL^®^ cuvette (101-QS) supplied by HELLMA^®^ (Müllheim, Germany).

#### 2.3.4. IR Spectroscopy

IR measurements were performed on an FTIR-Spectrometer Spectrum Two (Perkin Elmer, Rodgau, Germany). The samples were prepared as homogenous potassium bromide pellets. Spectra were obtained by PerkinElmer Spectrum following the instructions of the manufacturer. Wave number area: 8300–350 cm^−1^; spectral solution 0.5 cm^−1^; wave number accuracy better than 0.01 cm^−1^ at 3000 cm^−1^; wave number correctness: 0.1 cm^−1^ at 3000 cm^−1^; signal-to-noise-ratio: 9.300: 1 Peak to Peak, 5 s and 32,000: 1 Peak to Peak, 1 min.

#### 2.3.5. Quantification of Carboxyethyl Groups and Azide Groups of Modified Dextran

Quantification of carboxyethyl (CE) groups per dextran was performed as previously described by Schneider et al. using ^1^H-NMR spectroscopy, applying a method developed by Richter et al. [21,40]. Detection of N_3_-groups via IR spectroscopy and quantification via NMR-spectroscopy was performed as described by Schneider et al. [21].

### 2.4. Synthesis of Target Compounds

#### 2.4.1. Modification of Dextran

Dextrans of different molecular weights (10,000, 5000 and 1000) were modified to bear approximately two azide moieties per dextran molecule at the glucose repeating units and a free amine at the reducing end introduced via reductive amination as described by Schneider et al. [21]. Analytical data can be found in the Supplementary Materials (Figures S1–S6).

#### 2.4.2. Synthesis of BCN-IRDye700DX

To a solution of 10 mM NHS-activated IRDye700DX^®^ (LI-COR Biosciences, Lincoln, NE, USA) in DMSO, 2 eq of DIPEA and 1 eq of BCN-(PEG)2-amine was added and incubated for 3 h at RT in the dark. The reaction was quenched via the addition of 10 eq Tris. Due to the low volume of the reaction mixture, the reaction was performed in a PCR tube. Product formation was confirmed by ESI-MS analysis (Figure S7).

#### 2.4.3. Generation of Nanobody–Dextran Conjugates Labelled with Cy5 and IRDye700DX

A reaction mixture of NB (1 eq), 50–100 eq of N_3_-Dex-cad and 0.1 eq of microbial transglutaminase (mTG) in 1× PBS was incubated for 3 h at 30 °C. SDS-PAGE and HIC analysis was performed for reaction control (Figure S8). Subsequently, the conjugates were purified utilizing Ni-nitrilotriacetic acid (NTA) agarose resin (HisPur™ Ni-NTA Resin, Thermo Fisher Scientific) in a 2 mL syringe with frit, following the instructions of the supplier. After dialysis, a solution of Cy5-DBCO or BCN-IRDye700DX^®^, respectively, was added (1–4 eq of label/N_3_) and the reaction mixture was incubated for 48 h at 4 °C. Purification was performed utilizing Ni-NTA mag-beads (PureCube Ni-NTA MagBeads, Cube Biotech, Monheim, Germany) following the instructions of the supplier. The DOC of the final constructs was determined via UV/Vis spectroscopy.

#### 2.4.4. Generation of Directly Labelled IRDye700DX Nanobody Conjugates

For spheroid penetration and NB-PDT assays, NB_A_ and NB_BC_ were also directly labeled with IRDye700DX^®^ (LI-COR Biosciences, Lincoln, NE, USA) according to the manufacturer’s protocol and as previously described in [37]. Free unconjugated PS was removed using four consecutive Zeba Spin Desalting Columns (Thermo Fisher Scientific, Perbio Science Nederland, Etten-Leur, The Netherlands). Further characterization of the NB-PS conjugates was performed as previously described [27], yielding two conjugates, namely NB_A_-PS DOC 0.9 and NB_BC_-PS DOC 1.5 [28].

### 2.5. Cell Culture and Cell Assays

#### 2.5.1. Cell Culture and Spheroid Formation

The human epidermoid carcinoma cell line A431, the human cervical adenocarcinoma cell line HeLa and the human mammary adenocarcinoma cell line MCF-7 were purchased from the American Type Culture Collection (ATCC) and cultured in high-glucose Dulbecco’s Modified Eagle’s medium (DMEM) (Lonza, Basel, Switzerland) supplemented with 10% fetal calf serum (GE Healthcare Life Science, Uppsala, Sweden), 100 μg/mL streptomycin, and 100 U/mL penicillin (Invitrogen, Carlsbad, CA, USA) and were kept at 37 °C, ~21% O_2_, 5% CO_2_ in a humidified atmosphere.

To cultivate 3D spheroids from A431 cells, a thin layer of Matrigel (Corning, Bedford, MA, USA) was coated on the bottom of an 8-well plate (Nunc Lab-Tek II Chambered Slide 8-wells plates) and incubated at 37 °C for 20 min to allow polymerization. A suspension of 2 × 10^4^ cells was added per well and incubated at 37 °C for 6–7 days. Spheroids with a diameter of 150–200 µm were used for further experiments. The growth and size of the spheroids was monitored closely with an EVOSfl digital inverted microscope, and the medium was exchanged regularly.

#### 2.5.2. Binding Assays with Cells

To determine the apparent binding affinities (K_D_) of NB-conjugates, EGFR-expressing human cells were seeded to a 96-well plate (1 × 10^4^ cells per well) and incubated at 37 °C. The next day, the binding assay was performed as previously described by incubating the cells with a concentration range of the NB-conjugates for 2 h at 4 °C [26,35]. For NBs conjugated to IRDye700DX^®^ or Cy5, the fluorescence of bound NB conjugates was detected with an Odyssey infrared scanner (LI-COR, Lincoln, NE, USA) at 700 nm or 800 nm, respectively. Experiments were conducted in biological duplicates of technical triplicates. The data depicted show one technical triplicate being representative for the biological replicates.

#### 2.5.3. Treatment of Spheroids with Conjugates and Confocal Microscopy

In order to evaluate and compare the penetration ability and distribution of the different NB_A_ and NB_BC_ conjugates in an in vitro 3D model, a multicellular tumor spheroid assay was employed similar to what was described previously [28]. Spheroids were incubated with 40 nM of the NB-Cy5 and NB-PS conjugates for different time intervals at 37 °C protected from light. Following incubation, unbound conjugate was removed by washing the spheroids with DMEM and PBS followed by fixation with 4% paraformaldehyde (Merck, Darmstadt, Germany) supplemented with 0.1% glutaraldehyde in case of NB-Cy5 conjugates. Background fluorescence was quenched using 100 mM glycine (Sigma-Aldrich) and cells were permeabilized using 0.5% Triton X-100 (Sigma-Aldrich).

For photosensitizer conjugates, direct detection of the PS fluorescence was not possible; thus, the spheroids were blocked with 2% BSA in 1× PBS and NB-PS conjugates were detected by incubating spheroids with rabbit anti VHH antibody (QVQ) overnight at 37 °C. After removal of the unbound primary antibody, spheroids were incubated with goat anti rabbit IgG Alexa Fluor 555 (Invitrogen, Carlsbad, CA, USA) for 4 h at 37 °C. Finally, all spheroids were counterstained with DAPI (Roche, Mannheim, Germany) to visualize cell nuclei and the slides were mounted with Mowiol (Sigma-Aldrich). All steps were performed protected from light.

Imaging was performed using a confocal laser scanning microscope LSM700 (Carl Zeiss Microscopy GmbH, Oberkochen, Germany) with a × 40 oil objective (EC Plan-NeoFluar × 40/1.3 Oil DIC). Pictures of spheroids were taken through the middle z-stack section and analyzed as previously described using ImageJ 1.53e software and the radial profile angle plug-in [28]. A minimum of six spheroids from two independent experiments were analyzed per time point. Finally, the normalized radial fluorescence profile plots were used to calculate the area under the curve (AUC) and the resulting AUC values (±SD) were plotted as a function of time.

#### 2.5.4. Nanobody-Targeted Photodynamic Therapy (In Vitro) on 2D Monolayer Cell Culture

EGFR-positive cells exhibiting high (A431), medium-to-low (HeLa) or low (MCF-7) EGFR expression levels were utilized for NB-PDT assays, as previously described [27,35].

One day prior to the experiment, cells were seeded at 1 × 10^4^ cells per well to a 96-well plate and incubated at 37 °C overnight. When a cell confluency of 75% was reached, cells were incubated with a concentration range of NB-PS conjugate in DMEM without phenol red and L-glutamine-supplemented 10% FBS and Pen-Strep. Cells were treated in dim light and incubated in the dark for 30 min at 37 °C (also referred to as pulse). Unbound conjugate was removed and the plate was scanned with an Odyssey infrared scanner at 700 nm to detect cell-associated NB-PS.

The cells were irradiated immediately after at room temperature. A431 and MCF-7 cells were irradiated with a fluence rate of 5.1 mW/cm^2^ and HeLa cells with 7 mW/cm^2^ (measured with an Orion PD Optometer) using a custom-made device consisting of 96 LED lamps (±690 nm, 1 LED per well). A431 and MCF-7 cells were illuminated for 33 min for a total light dose of 10 J/cm^2^, while HeLa cells were illuminated for a total of 60 min to add up to a total light dose of 25 J/cm^2^. Non-irradiated control plates were treated similarly, except cells were placed back into the incubator immediately after the pulse scan.

After irradiation, plates were kept in the dark at 37 °C for 24 h before performing a cell viability assay using Alamar Blue reagent (Bio-Rad) according to the manufacturer’s instructions. To control for 0% viability of cells, cells were exposed to 1% TritonX-100 and cell death was monitored using an EVOSfl microscope. Fluorescence was measured with a FLUOstar Optima microplate reader (BMG Labtech) and results expressed as cell viability in percentage relative to untreated cells. The median effective concentration (EC50), i.e., concentration of conjugate to achieve 50% of cell death, was determined using GraphPad Prism using a log (inhibitor) vs. normalized response fit.

#### 2.5.5. NB-Targeted PDT on 3D Spheroid Cell Culture and Cell Viability Assay

On the day of treatment, spheroids were incubated with NB-PS conjugates (25, 50 and 100 nM in duplicates) in DMEM without phenol red (+10% FBS and Pen-Strep) for 2 h at 37 °C in the dark. Immediately after removing unbound conjugate, spheroids were irradiated at room temperature with a total light dose of 20 J/cm^2^ (±690 nm, fluence rate of 7 mW/cm^2^ confirmed by an Orion PD Optometer, total illumination time of 48 min). Subsequently, two untreated wells were exposed to 1% TritonX-100, serving as a control for 0% viability, and spheroids were incubated for 24 h at 37 °C. Bright field images of spheroids 24 h post NB-targeted PDT were taken on an EVOSfl or Nikon Eclipse digital inverted microscope using a 20× objective.

The next day, spheroid-containing microtiter plates were incubated with CellTiter-Glo 3D reagent (Promega, Madison, WI, USA) according to the manufacturer’s instructions. Thereafter, the contents of the microtiter plate were transferred to a white 96-well plate (Greiner Bio-One, Alphen a/d Rijn, The Netherlands) and the luminescence signal was measured using a GlowMax Luminometer (Promega).

Experiments were conducted in biological duplicates.

## 3. Results and Discussion

### 3.1. Generation of Compounds

The anti-EGFR-targeting nanobody NB_A_ was described recently to display an affinity to EGFR in the single-digit nanomolar range even after conjugation with a PS [35]. 7D12-9G8 (renamed NB_BC_) is a fusion protein of two anti-EGFR nanobodies that bind at different sites of the EGFR extracellular domain showing biparatopic binding with single-digit nanomolar affinity for the NB_BC_ [36], as well as in a NB_BC_-PS format [27].

**Figure 2:**
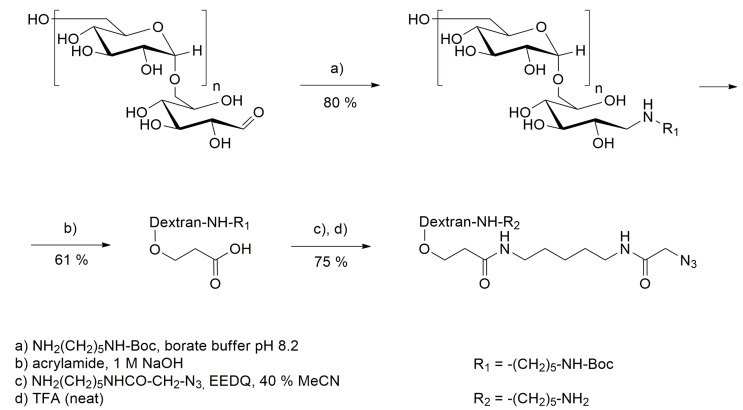
Synthesis route for the modification of dextran. EEDQ: N-ethoxycarbonyl-2-ethoxy-1,2-dihydroquinoline.

To both nanobodies, a His_6_-tag was introduced *N*-terminally for purification purposes. In parallel, to enable the attachment of the dextran polymer, the C-terminus of both nanobodies was extended by a microbial transglutaminase (mTG) recognition sequence, SPI7G, for enzyme-mediate polymer coupling as described previously [21]. For attachment of payload to dextran via SPAAC and also to facilitate mTG-mediated conjugation of the polysaccharide to the nanobodies, dextran was modified as described by Schneider et al. [21]. Synthesis started with reductive amination of the reducing end to introduce a Boc-protected cadaverine linker to enable mTG-mediated conjugation to nanobodies at later stages (Figure 2). Subsequently, carboxyethylation of that dextran-*N*-Boc-cadaverine (Dex-*N*-Boc-Cad) at the C2 hydroxy group of the glucose-units of dextran was performed. Hence, acrylamide was introduced upon nucleophilic conjugate addition and subsequently hydrolyzed to obtain carboxyethylated dextran-*N*-Boc-cadaverine (2-CED-Dex-*N*-Boc-Cad), comprising defined numbers of CE-groups.

To facilitate the decoration of the polysaccharide scaffold with desired cargos via SPAAC, a cadaverine–azide linker was introduced to the carboxy moieties of 2-CED-Dex-N-Boc-Cad upon amide bond formation, followed by deprotection of the Boc-group to obtain azido-functionalized dextran-cadaverine (N_3_-Dex-Cad) (Figure 2). Dextrans were modified to bear approximately two azide moieties per dextran. To examine the impact of size of the polysaccharide scaffold on the tumor spheroid penetration of dextraknobs, dextrans with molecular weights of 10,000, 5000 and 1000 were utilized.

**Figure 3:**
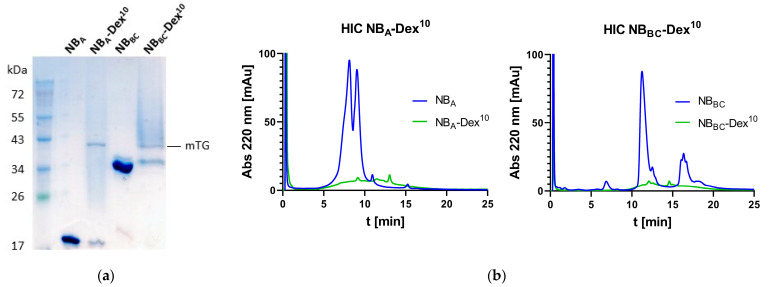
Analysis of nanobody dextran conjugates. (**a**) SDS-Gel of dextraknobs comprising NB_A_ and NB_BC_ as targeting protein before IMAC. The Coomassie smears at lane 2 and 4 indicate successful generation of dextraknobs. (**b**) HIC chromatograms of nanobodies and corresponding dextran conjugates.

NB-dextran conjugates were generated by mTG-mediated conjugation of N_3_-Dex-Cad and His-NB_A_ or His-NB_BC_, respectively (Figure 3a). Excess reactants and mTG were removed via immobilized metal ion chromatography (IMAC). Sodium dodecyl sulfate polyacrylamide gel electrophoresis (SDS-PAGE) and hydrophobic interaction chromatography (HIC) analysis were performed for reaction control. SDS-gels of the conjugates revealed a smear above the band of remaining unmodified NBs, which is attributed to NB-dextran conjugates (Figure 3a and Figure S8A–D,F) [21]. HIC analysis confirmed a quantitative conversion of the nanobodies (Figure 3b and Figure S8E) as reactant peaks of the unconjugated nanobodies diminish and a broad peak could be observed, which is caused by conjugation with dextran [21].

Subsequently, payload was attached to the NB-dextran conjugates via SPAAC. Therefore, DBCO-Cy5 and BCN-IRDye700DX^®^, which was readily produced prior to SPAAC from IRDye700DX^®^-NHS ester and BCN-PEG-amine, were added to the solution and incubated for 24 h at 10 °C. Excess reactant was removed via IMAC. DOCs of the generated fluorophore and PS-bearing conjugates were determined by UV/Vis-spectroscopy. All dextraknobs appeared to bear approximately one cargo molecule per conjugate, the highest achieved DOC being 1.2 PS per dextraknobs for the NB_BC_-Dex^10^ PS conjugate (Table 1).

A higher DOC could possibly not be reached in the conditions tested here due to steric hindrance caused by interactions of dextran with the conjugated nanobody and hence limited availability of azide moieties for SPAAC. Thus, decoration of dextran with a cargo of desire previous to conjugation with the protein of interest could be beneficial to reach higher DOCs. For this, a preferable cargo to test would be accessible in larger amounts and of smaller size. In the context of PSs, photoreactive agents derived from organo-metal complexes could be promising alternatives [41,42]. Along that, the utilization of more hydrophilic cargos would be preferable, but not crucial as it was shown previously that the hydrophilic dextran scaffold compensates the impact of the highly hydrophobic payload MMAE to the final construct [21]. Secondly, the utilization of a different enzyme should totally exclude the risk of crosslinking by rather promiscuous mTG, which was observed during the first experimental set ups of dextran conjugation through the more universal method. Recently, lipoic acid ligase (LplA) mutant W37V was utilized to introduce a moiety enabling click chemistry for further attachment of cargo. As LplAW37V was engineered to be highly specific towards its substrates, no side reaction and crosslinking should occur [43]. Furthermore, dextran could easily be modified to bear the corresponding counterpart for clicking it with the functionalized protein of interest.

### 3.2. Cell Binding Studies

**Figure 4:**
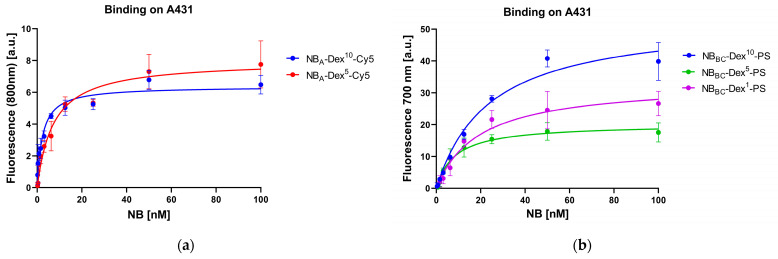
Cell binding studies of nanobody dextran conjugates. (**a**) Cell binding of Cy5-labeled conjugates on A431 cells. (**b**) Cell binding of PS-labeled conjugates on A431 cells. Data shown in this figure are derived from technical triplicates.

The apparent binding affinities (K_D_) of NB_A_-Dex^10^-Cy5 and NB_A_-Dex^5^-Cy5 conjugates binding to EGFR on the surface of A431 cells were found to be in the single-digit nanomolar range (Figure 4a) which is in line with published K_D_ values obtained for NB_A_ conjugated to PS [35]. These results indicate that binding properties were not impaired by the added dextran and Cy5 label.

The binding of NB_BC_-Dex-PS conjugates to EGFR was tested on A431 and on HeLa cells with comparatively moderate to low EGFR expression levels (Figure 4b and Figure S9 and Table S3). Apparent binding affinities of NB_BC_-Dex-PS variants were generally within the double-digit nanomolar range and therefore higher than the previously published binding affinities of NB_A_-PS and NB_BC_-PS [27,35]. NB_BC_-Dex^10^-PS exhibited the highest K_D_ on both A431 and HeLa cells, with 23 and 24 nM, respectively. For NB_BC_-Dex^1^-PS, K_D_s of 19 nM (A431) and 14 nM (HeLa) were observed as well as the lowest K_D_s of 8.4 nM on A431 and 8.7 nM on HeLa for NB_BC_-Dex^5^-PS. All NB_BC_-Dex-PS conjugates showed no significant binding on EGFR^-^ MCF-7 cells (Figure S10), which served as negative control.

### 3.3. D Penetration Studies

Since nanobodies’ small size is expected to be beneficial for efficient tissue distribution, here the different size NB_A_-Dex-Cy5 dextraknobs were tested for their penetration in 3D spheroids. For this purpose, a 3D tumor spheroid model of A431 was employed, similar to what was previously described by Beltrán Hernández et al. for Alexa Fluor 647-labeled nanobodies (Figure S11) [28].

**Figure 5:**
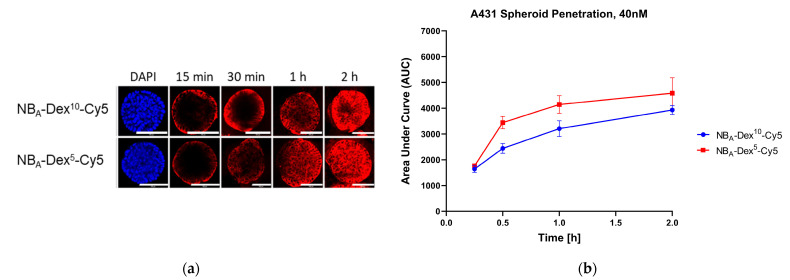

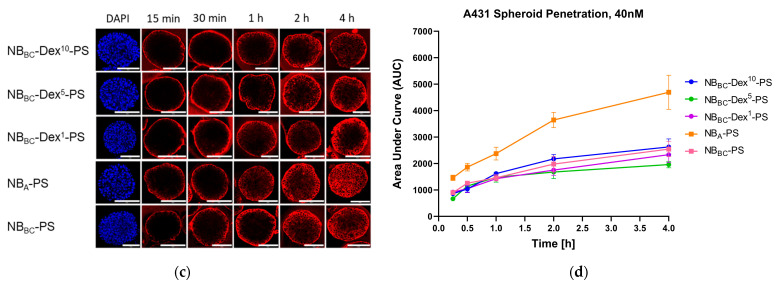
Three-dimensional penetration studies. (**a**) Confocal fluorescence microscopy images of Cy5-labeled dextraknobs. Scale bar: 100 µm. (**b**) AUC graph of Cy5-labeled dextraknobs. (**c**) Confocal fluorescence microscopy images of PS conjugates. Scale bar: 100 µm. (**d**) AUC graph of PS conjugates.

Both conjugates were observed to distribute towards the spheroid center over time, as indicated by the significant increase in fluorescent signal from the outer rim towards the core of the spheroid with time (Figure 5a). The fluorescence signal of NB_A_-Dex-Cy5 conjugates was detected in the center of spheroids 15–30 min after nanobody addition and this signal increased over time until reaching relatively similar values as the rim after 2 h for conjugate with a dextran of MW 5000.

For NB_A_-Dex^10^-Cy5, fluorescence intensities were generally observed to be lower in the spheroid core compared to the outer lining after 2 h, which is also visualized well by the normalized intensity profile plots of the tracers along the radius of the spheroids (Figure S12). From these normalized radial intensity profiles, the area under the curve (AUC) was determined and plotted against time (Figure 5b). Some delay in compound distribution in the spheroids was observed for NB_A_-Dex^10^-Cy5, in comparison with NB_A_-Dex^5^-Cy5. The differences in penetration within the timepoints were found to be significant when using an ordinary one-way ANOVA as indicated by one asterisk (*p* ≤ 0.05) starting from 30 min (30 min: *p* = 0.030, 1 h: *p* = 0.046, 2 h: *p* = 0.032). Thus, it was concluded that conjugates equipped with a dextran of a molecular weight of 5000 show better penetration behavior than conjugates with MW 10,000 dextran.

Concerning NB_BC_-Dex-PS and NB-PS conjugates, after one hour, the fluorescence signal of all conjugates was clearly visible closer to the center of the spheroids (Figure 5c). However, except for NB_A_-PS, the detected fluorescence in the core of the spheroids stayed lower than the outer rim and did not reach similar signal values for all conjugates within 4 h. Comparison of spheroid distribution and AUC of both NB_A_-Dex-Cy5 conjugates with NB_A_-PS (Figure 5d) seems to be similar for these constructs, the NB_A_-Dex^5^-Cy5 conjugate showing even faster distribution than the NB_A_-PS conjugate. This might be attributed to the MW 5000 dextran compensating the contribution of the hydrophobic PS and therefore maintaining overall hydrophilicity of the construct sufficiently. On the other hand, the MW 10,000 dextran compensates for the PS’s hydrophobicity, but further increases construct size and therefore increases penetration time, resulting in no significant change in penetration behavior compared with NB_A_-PS. Similarly to the comparison with NB_A_-PS, penetration behavior of NB_A_-Dex-Cy5 conjugates was comparable to the distribution of a 7D12-Alexa647 conjugate observed before [28], where the core of the spheroid was reached after 2 h, further suggesting that conjugation of a monomeric NB to a dextran scaffold only has a minor impact to the penetration behavior.

Furthermore, the obtained AUC graph of PS conjugates demonstrates a generally delayed or different penetration behavior of all NB_BC_ conjugates compared to the NB_A_ conjugate, indicated by the smaller slope over time which was similar for all NB_BC_ conjugates, which meets observations of previous studies [27,28,44]. ANOVA analysis indicated by three to four asterisks and post hoc Tukey test group pairings implied that variations within NB_BC_ conjugate pairs were insignificant and negligible.

Focusing on contribution by the utilized nanobodies to penetration behavior, group pairings confirmed that significance was indeed related to penetration of the NB_A_-PS conjugate in comparison with NB_BC_ conjugates. This could be related to the larger size of the biparatopic NB_BC_, but also biparatopic binding resulting in a lower apparent K_D_ of the parental biparatopic NB could lead to stronger immobilization of constructs at cells of the outer rim and internalization due to clustering of the NB_BC_ [27], therefore preventing constructs from faster penetration [28,36,44,45,46]. Although NB_BC_-Dex-PS conjugates did not show significant differences in penetration behavior to each other, surprisingly the AUC graph indicated that NB_BC_-Dex^10^-PS penetrated slightly better compared to other biparatopic dextran conjugates. Following the argumentation of previous section, this could be caused by hydrophobicity of the PS, which would be better compensated by dextran of higher molecular weight and in case of less compensation would lead to higher stickiness of the conjugate and thus worsening penetration behavior. Additionally, dextran could hinder binding of the nanobody due to steric hindrance to some extend as was observed for engineered T-cell engagers equipped with XTEN polypeptide masks at the N- and C-terminus, targeting a tumor antigen (human epidermal growth factor receptor 2 (HER2) or epidermal growth factor receptor (EGFR)) and CD3 [47]. These constructs showed 4-log-fold protection of binding caused by steric hindrance by the XTEN masks. Therefore, larger dextran could have a higher impact on hampering antigen binding of the targeting NB_BC_ than a polysaccharide of smaller size, leading to less immobilization of the conjugate at the cell surface and thus faster penetration through the tumor.

### 3.4. Nanobody-Targeted PDT Studies

**Figure 6:**
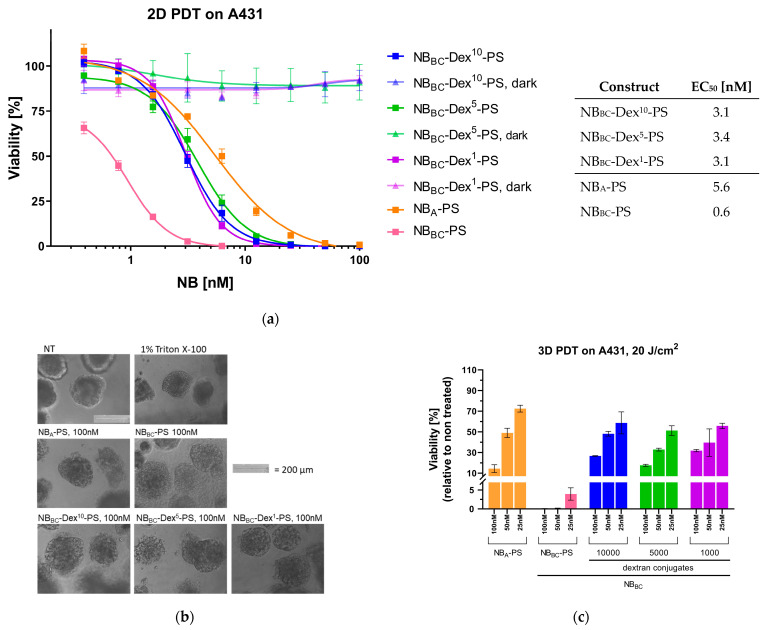
Nanobody PDT studies. (**a**) Cell viability (%) after a 10 J/cm^2^ light dose relative to untreated cells and corresponding EC_50_ values. Data shown are derived from technical triplicates. (**b**) Bright-field images of A431 spheroids treated with NB-PDT, 24 h after irradiation. Scale bar: 200 µm. (**c**) 3D NB-targeted PDT. Viability (%) of A431 spheroids relative to untreated spheroids after treatment with 3 different concentrations of the NB-PS for 2 h followed by exposure to a 20 J/cm^2^ light dose (mean ± SD). Data shown are derived from technical triplicates.

To further explore the potential of dextraknobs for EGFR-targeted nanobody photodynamic therapy, an in vitro assay was performed on a monolayer of A431 and MCF-7 cells as previously described with NB_BC_-PS conjugates [27]. First of all, treatment of MCF-7 with dextran conjugates did not affect viability (Figure S13), even if the fluorescence signal observed after pulse (Figure S14) was slightly higher than what was seen in the previous binding assay. When omitting illumination, the dextran conjugates applied on A431 cells also did not affect cell viability and EC50 values could not be determined, verifying the specific activation of the PS by illumination (Figure 6a). Moreover, it can be assumed that there is no cytotoxicity related to the dextran modification of the nanobodies, which was expected for the FDA-approved polysaccharide broadly applied for generation of prodrugs [48].

Clearly, NB_BC_-PS with a DOC 1.5 was most potent with an EC_50_ value of only 0.6 nM on A431 cells which was in line with the efficacy observed with the same conjugate under illumination in a previous study [27]. Interestingly, the efficacy of NB_BC_-Dex-PS conjugates were very similar with half-maximal effective concentrations of around 3 nM for all three conjugates, which did not correlate with the differences in maximal fluorescence observed after pulse (Figure S15). Additionally, EC_50_ values were in a low nanomolar range and even slightly lower than the monomeric NB_A_-PS, further supporting the potency of the biparatopic format [27] and verifying the three different NB_BC_-Dex-PS dextraknobs can deliver PS similarly in these cultures. How the phototoxic effect measured in the Alamar and CellTiter-Glo assay eventually translates into reduced tumor growth will require further evaluation in animal studies.

Encouraged by the potency of NB_BC_-Dex-PS conjugates in 2D targeted PDT, a following PDT assay was performed using a 3D spheroid model. For this purpose, A431 spheroids were treated with different concentrations of NB conjugates for 2 h at 37 °C (referred to as pulse) and exposed to a light dose of 20 J/cm^2^. Immediately after irradiation, the morphology of NB-treated spheroids appeared visibly changed and disrupted compared to non-treated spheroids. The following day, the morphology of all spheroids treated with NB_BC_-PS appeared to be most disrupted compared to spheroids treated with the other conjugates (Figure 6b). Additionally, the morphology of spheroids treated with NB_A_-PS and the dextran conjugates NB_BC_-Dex-PS seemed to have changed slightly over 24 h, as displayed by the more compact appearance.

Clearly, viability was affected the most by treatment of spheroids NB_BC_-PS, highlighting the potency of higher prodrug payload once more (Figure 6c). When treated with NB_BC_-PS, a lethality of approximately 100% was observed from 100 nM down to 25 nM, Treatment with 25 nM resulted in a mean viability of approximately 4%.

Similar to observations made in the 2D targeted PDT assay, viabilities of spheroids treated with dextran conjugates of NB_BC_ were all similar and comparable to NB_A_-PS, although the morphology of spheroids 24 h post irradiation appeared more disrupted for NB_BC_-Dex-PS conjugates (Figure S16), despite variations in accumulation within 2 h observed earlier. The more disrupted appearance of the spheroids could suggest more damage to the outer cell layers of the 3D culture. Moreover, no significant difference in cell viability could be observed in regard to dextran size or DOC. These results encourage the further exploration of the generation of dextraknobs, possibly with higher DOC (i.e., 3–6).

## 4. Conclusions

In summary, we reported the successful generation of nanobody–dextran conjugates, so called dextraknobs, for application in targeted approaches for treatment and imaging of cancerous tissues. Thus, Cy5-labeled dextraknobs comprising the anti-EGFR nanobody NB_A_ as targeting protein showed a distribution in A431 tumor spheroids comparable to the nanobody directly labeled with a PS. Additionally, the utilization of smaller sized dextran (MW 5000 vs. 10,000) seemed to be beneficial for more homogeneous spheroid penetration in this format. Applying the biparatopic NB_BC_ as targeting molecule, penetration behavior of dextraknobs comprising dextran with an MW of 1000, 5000 and 10,000 labeled with PS IRDye700DX^®^ was again comparable to a NB_BC_-PS conjugate with DOC 1.5. However, NB_A_-PS showed deeper and more homogenous tumor spheroid penetration than all NB_BC_ conjugates, confirming that the utilization of only one VHH would be more favorable in terms of distribution than the biparatopic format [26,28].

Investigation of the potency of PS-labeled constructs with DOC ~ 1 in 2D PDT studies revealed efficient cell killing by all dextraknobs in the single-digit nanomolar range, which was in line with efficacy of the tested NB_A_-PS conjugate. Additionally, a correlation between higher DOC and potency was shown by sub-nanomolar EC50 values for NB_BC_-PS. This trend was confirmed in 3D PDT studies on A431 spheroids. Taken together, the generation of dextraknobs with DOC > 2 could possibly lead to highly potent PDT. For that, several modifications in generation strategy could be beneficial to increase DOC of dextraknobs. Thus, since the distribution of dextraknobs in 3D spheroids is not compromised by the MW of dextran here tested, we are encouraged to examine this approach further, also considering different target and cancer types.

## Figures and Tables

**Table 1:** Generated conjugates and corresponding DOC values.

**Abbreviation**	**NB**	**Dextran**	**DOC**	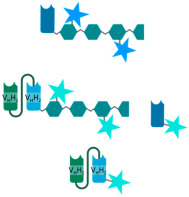
NB_A_-Dex^10^-Cy5	NB_A_	10,000	0.7	
NB_A_-Dex^5^-Cy5	NB_A_	5000	1	
NB_BC_-Dex^10^-PS	NB_BC_	10,000	1.2	
NB_BC_-Dex^5^-PS	NB_BC_	5000	0.7	
NB_BC_-Dex^1^-PS	NB_BC_	1000	1.1	
NB_A_-PS	NB_A_	-	0.9	
NB_BC_-PS	NB_BC_	-	1.5	

## Data Availability

Not applicable.

## Supplementary Materials

The following supporting information can be downloaded at https://www.mdpi.com/article/10.3390/pharmaceutics15102374/s1, Scheme S1: Synthesis of the N_3_-Cad-Linker (N-(aminopentyl)-2-azidoacatamide); Scheme S2: Reductive amination of dextran with *N*-Boc-cadaverine; Scheme S3: Carboxyethylation of Dex-*N*-Boc-Cad to 2-CED-*N*-Boc-Cad; Scheme S4: N_3_-functionalization of 2-CED-*N*-Boc-Cad towards N_3_-Dex-Cad; Scheme S5: Synthesis of BCN-IRDye700DX^®^; Scheme S6: Structural formulas of utilized labels; Figure S1: HPLC-diagram of the N_3_-Cad-Linker (0 to 80% Eluent B, 220 nm); Figure S2: LC-MS spectrum of the N_3_-Cad-Linker (exact mass = 185.13 *m*/*z*); Figure S3: ^1^H-NMR spectra of Dex-*N*-Boc-Cad variants; Figure S4: ^1^H-NMR spectra of 2-CED10-*N*-Boc-Cad variants; Figure S5: ^1^H-NMR spectra of N_3_-Dex-Cad variants; Figure S6: IR-Spectra of N_3_-Dex^10^-Cad variants; Figure S7: ESI-MS spectrum of BCN-IRDye700DX^®^; Figure S8: (A–D): Coomassie stained SDS-Gels of generated dextraknobs. (E): HIC chromatogram of NB_A_-Dex10. (F): SDS-Gel imaged with fluorescence reader (700 nm). Bands show excited IRDye700DX^®^; Figure S9: Data of binding assay of NB-PS conjugates on HeLa cell line; Figure S10: Data of binding assay of NB_BC_-Dex-PS conjugates on MCF-7 cells; Figure S11: Schematic representation of the analysis of confocal images taken of spheroids; Figure S12: Normalized integrated intensity plots of all conjugates at time points 15 min, 30 min, 1 h and 2 h. PS- conjugates were also measured at 4 h; Figure S13: Percentages (%) of cell viability after a 10 J/cm^2^ light dose relative to untreated cells; Figure S14: Fluorescence intensities of NB_BC_-Dex-PS conjugates bound to cells after 30 min pulse incubation with a concentration range of the conjugates; Figure S15: Fluorescence intensities of NB-PS conjugates bound to cells after 30 min pulse incubation with a concentration range of the conjugates; Figure S16: 3D PDT 20J/cm^2^, phase-contrast microscopy images taken 24 h post treatment/irradiation; Table S1: Calculated CE-ratios per dextran; Table S2: Yields of N_3_-functionalization of 2-CED-*N*-Boc-Cad with the N_3_-cad-linker and determined number of N_3_-groups per dextran (by ^1^H-NMR); Table S3. K_D_-values of tested conjugates on HeLa cells.
