# Supplementary material for: Penetration of Nanobody-Dextran Polymer Conjugates through Tumor Spheroids
Source: Pharmaceutics. 2023 Sep 22;15(10):2374. doi: 10.3390/pharmaceutics15102374 (PMC10609859; doi:10.3390/pharmaceutics15102374)
Supplement: Supplementary file 1 [file pharmaceutics-15-02374-s001.zip › pharmaceutics-2617131-supplementary.pdf]

Supplementary information

Penetration of Nanobody-Dextran Polymer Conjugates through Tumor Spheroids

Peter Bitsch [1,†], Eva S. Baum [2,†], Irati Beltrán Hernández [2,3], Sebastian Bitsch [1], Jakob Harwood [1], Sabrina Oliveira [2,3,*], and Harald Kolmar [1,4,*]

[1] Institute for Organic Chemistry and Biochemistry, Technical University of Darmstadt, Peter-Grünberg-Str. 4, 64287 Darmstadt, Germany
[2] Cell Biology, Neurobiology and Biophysics, Department of Biology, Faculty of Science, Utrecht University, Padualaan 8, 3584 CH Utrecht, The Netherlands
[3] Pharmaceutics, Department of Pharmaceutical Sciences, Faculty of Science, Utrecht University, Universiteitsweg 99, 3584 CG Utrecht, The Netherlands
[4] Centre of Synthetic Biology, Technical University of Darmstadt.
* Correspondence: s.oliveira@uu.nl (S.O.); harald.kolmar@tu-darmstadt.de (H.K.)
† These authors contributed equally to this work.

Table of contents

1. Synthesis of compounds 2
1.1. Synthesis of cadaverine-azide linker 2
1.2. Synthesis of dextran-*N*-Boc-cadaverine 3
1.3. Carboxyethylation of dextran 4
1.4. Synthesis of N_3-dextran-*N*-Boc-cadaverine 6
1.5. Synthesis of BCN-IRDye700DX 9
1.6. Structural formulas of utilized labels 10
1.7. Generation of dextraknobs 11
2. Supplementary figures cell assays 12
2.1. Cellular binding assays 12
2.2. Confocal microscopy of spheroids 13
2.3. .... Nanobody-targeted photodynamic therapy (in vitro) on 2D monolayer cell culture 14
2.4. NB-targeted PDT on 3D spheroid cell culture 16

1. Synthesis of compounds

1.1. Synthesis of cadaverine-azide linker

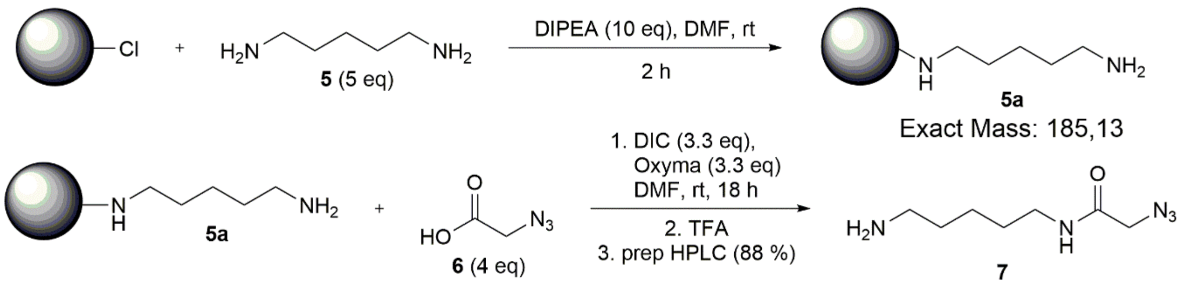

Scheme S 1. Synthesis of the N_3-Cad-Linker (*N*-(aminopentyl)-2-azidoacatamide).

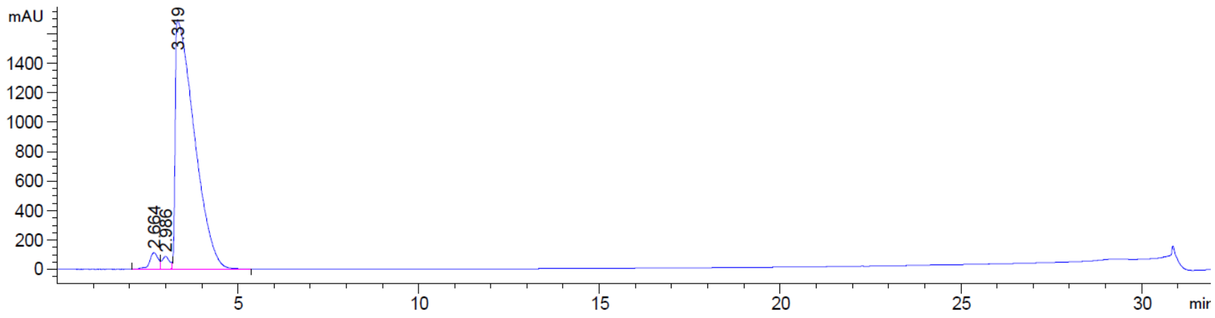

Figure S 1. HPLC-diagram of the N_3-Cad-Linker (0to80 % Eluent B, 220 nm).

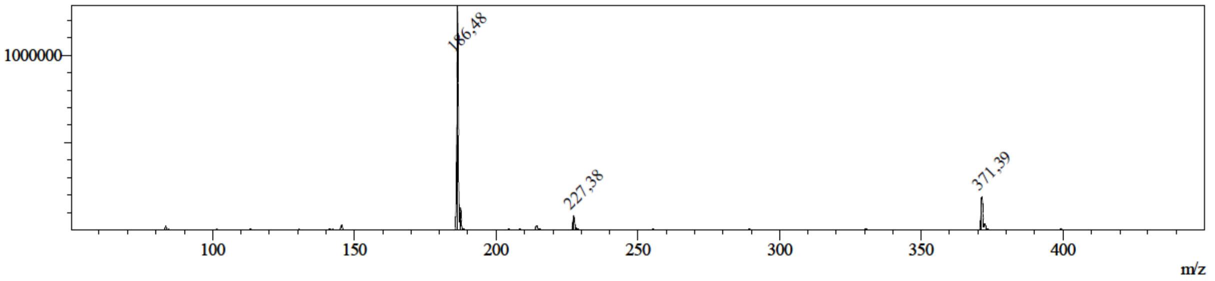

Figure S 2. LC-MS spectrum of the N_3-Cad-Linker (exact mass = 185.13 m/z). As method, a gradient from 10to100 % Eluent B was used over 20 min with a flow rate of 0.7 mL/s.

1.2. Synthesis of dextran-*N*-Boc-cadaverine

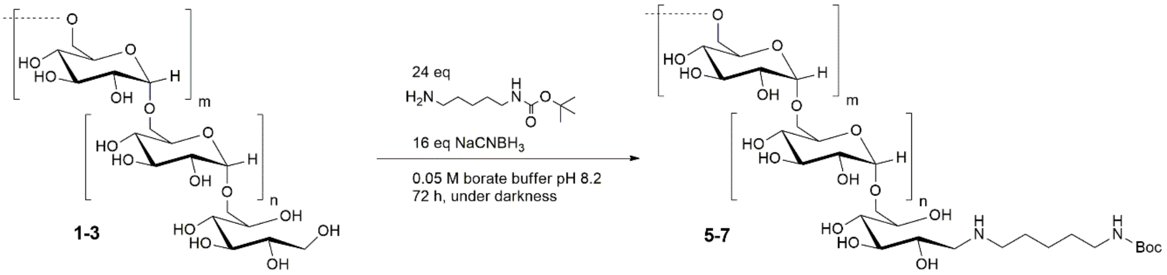

Scheme S 2. Reductive amination of dextran with *N*-Boc-cadaverine.

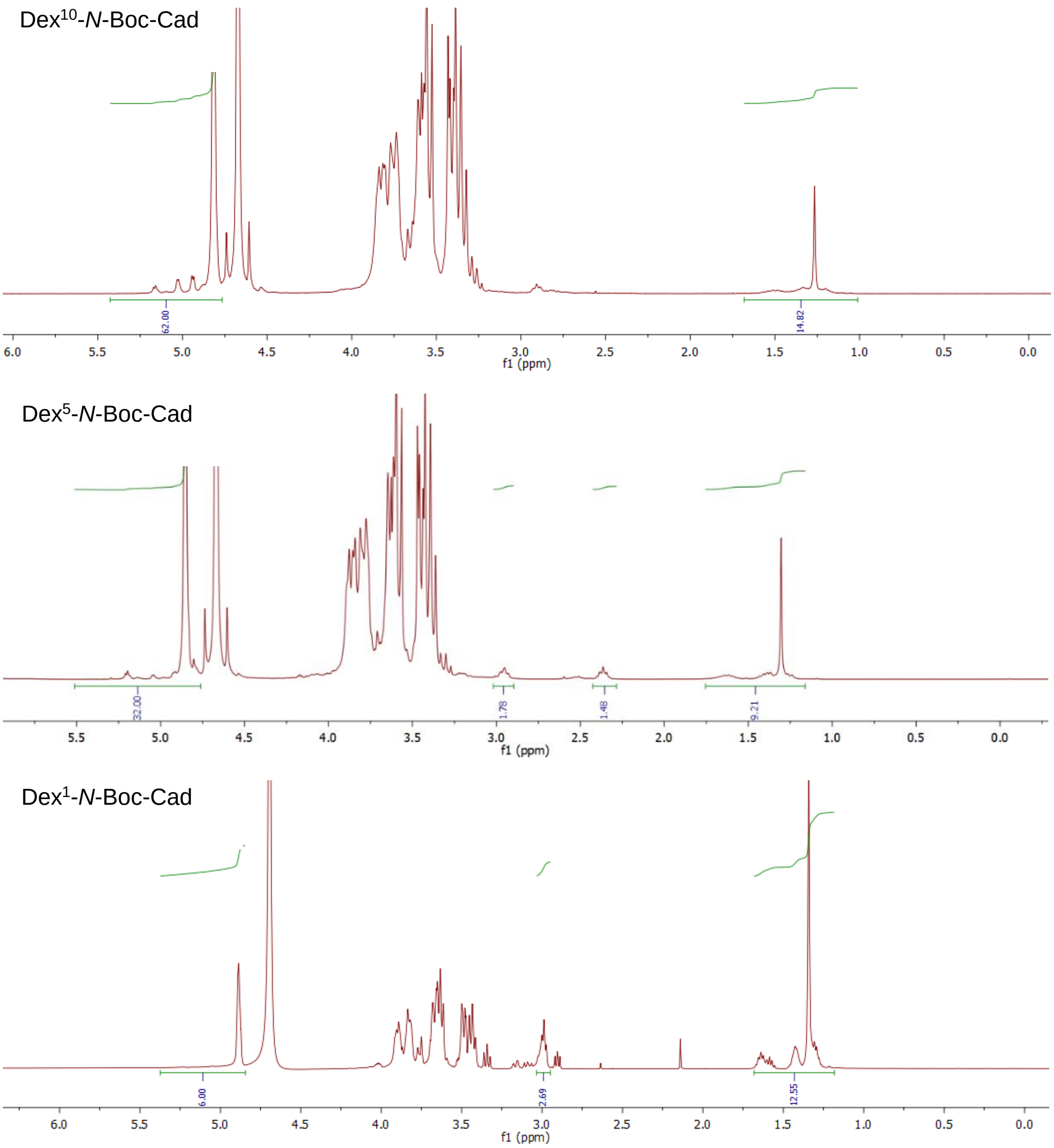

Figure S 3. ^{1}H-NMR spectra of Dex-*N*-Boc-Cad variants. ^{1}H-NMR (300 MHz, deuterium oxide) δ = 5.36 – 4.80 (m, 1 H, C(1)H), 4.20 – 3.22 (m, C(2-6)H (glucose units)), 3.05 – 2.81 (m, 4 H, C**H**$_2$NH-Boc, C**H**$_2$(CH$_2$)$_4$NHBoc), 1.73 – 1.19 (overlapped m, 15H, C**H**$_2$(CH$_2$)$_3$NH-Boc, C**H**$_2$(CH$_2$)$_2$NH-Boc, C**H**$_2$(CH$_2$)$_1$NH-Boc, 3 C**H**$_3$(Boc)) ppm.

1.3. Carboxyethylation of dextran

Table S 1. Calculated CE-ratios per dextran.

compound	CE-groups per dextran	Yield [%]
2-CED[10]-*N*-Boc-Cad	2.4	65
2-CED[5]-*N*-Boc-Cad	2.4	73
2-CED[1]-*N*-Boc-Cad	4.3	45

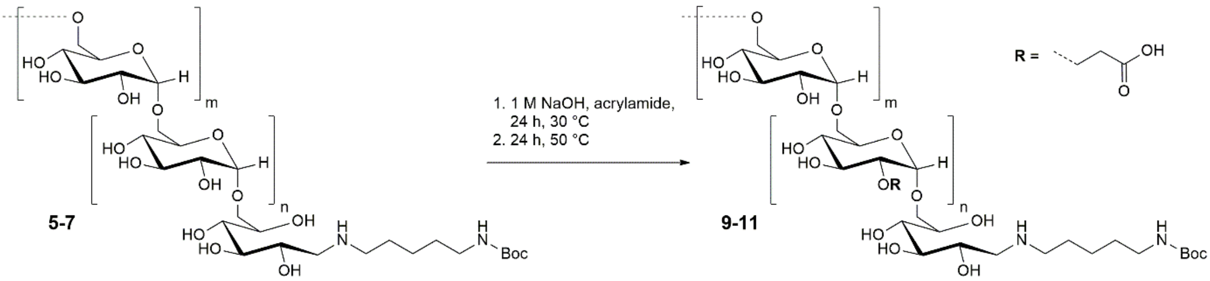

Scheme S 3. Carboxyethylation of Dex-*N*-Boc-Cad to 2-CED-*N*-Boc-Cad.

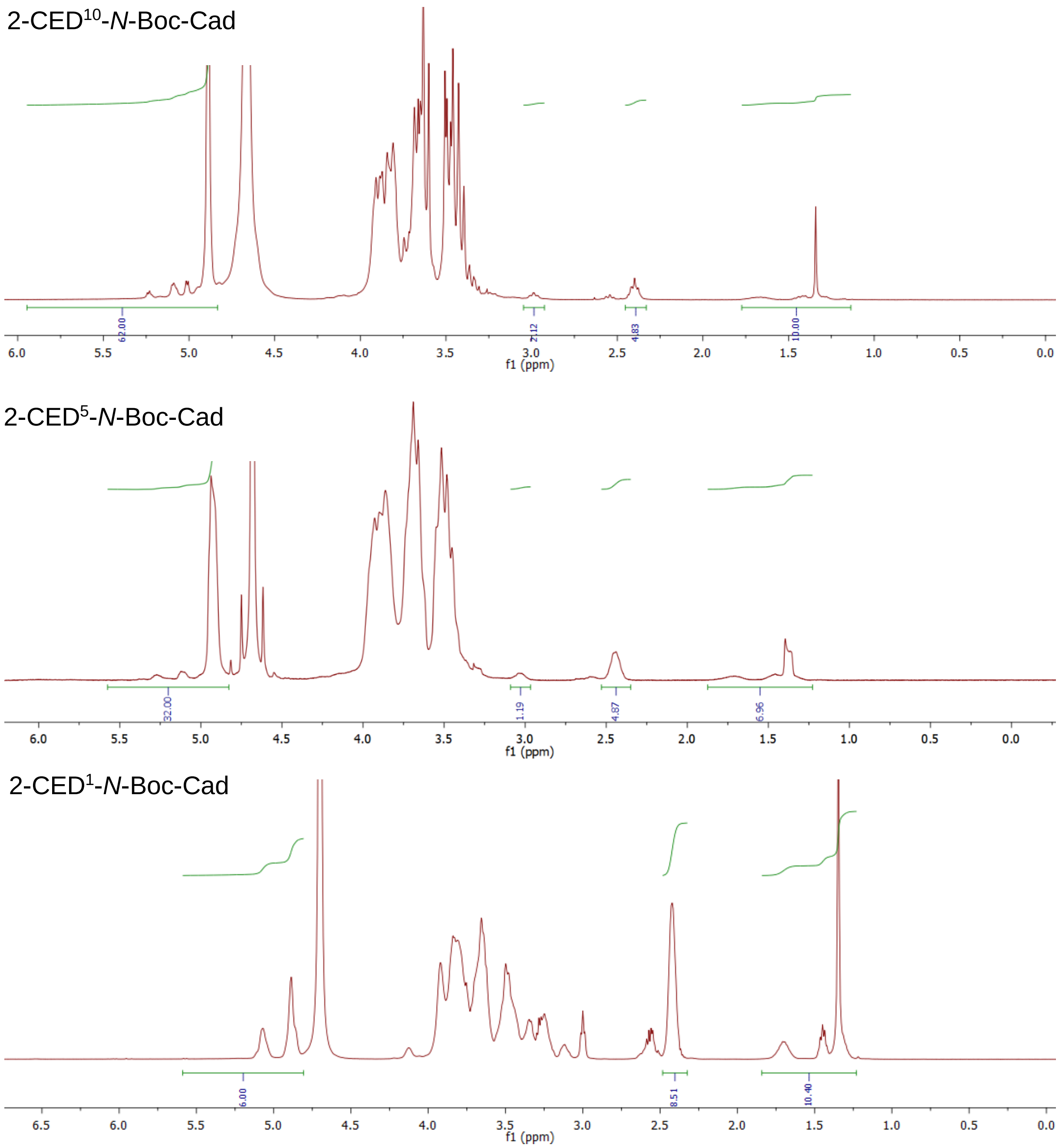

Figure S 4. ^{1}H-NMR spectra of 2-CED^{10}-*N*-Boc-Cad variants. ^{1}H-NMR of CE-dextranes (300 MHz, D_2O) δ = 6.09 – 4.79 (m, 1 H, C(1)H), 4.34 – 3.16 (m, C(2-6)H (glucose units); C**H**$_2$CH$_2$COOH), 3.14 – 2.91 (m, 4 H, C**H**$_2$NH-Boc, C**H**$_2$(CH$_2$)$_4$NH-Boc), 2.52 – 2.29 (t, C**H**$_2$COOH), 1.82 – 1.24 (overlapped m, 15 H, C**H**$_2$(CH$_2$)$_3$NH-Boc, C**H**$_2$(CH$_2$)$_2$NH-Boc, C**H**$_2$(CH$_2$)$_1$NH-Boc, 3 C**H**$_3$(Boc)) ppm.

1.4. Synthesis of N_3-dextran-*N*-Boc-cadaverine

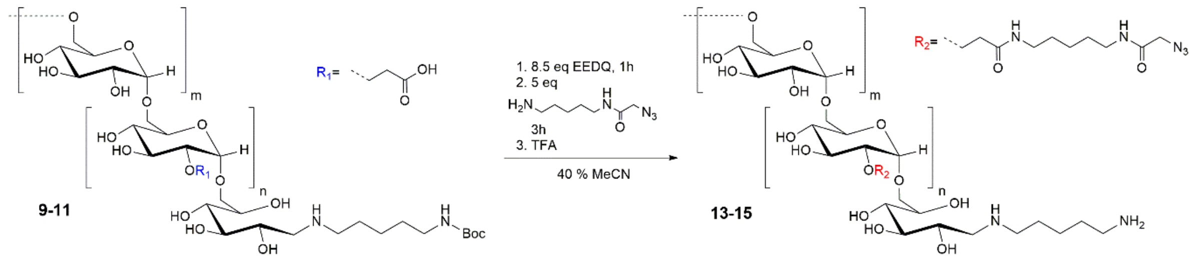

Scheme S 4. N_3-functionalization of 2-CED-*N*-Boc-Cad towards N_3-Dex-Cad.

Table S 2. Yields of N_3-functionalization of 2-CED-*N*-Boc-Cad with the N_3-cad-linker and determined number of N_3-groups per dextran (by ^{1}H-NMR).

compound	N_3-groups per dextran	Yield [%]
N_3-Dex^{10}-Cad	2.4	70
N_3-Dex^{5}-Cad	2.4	65
N_3-Dex^{1}-Cad	4.3	40

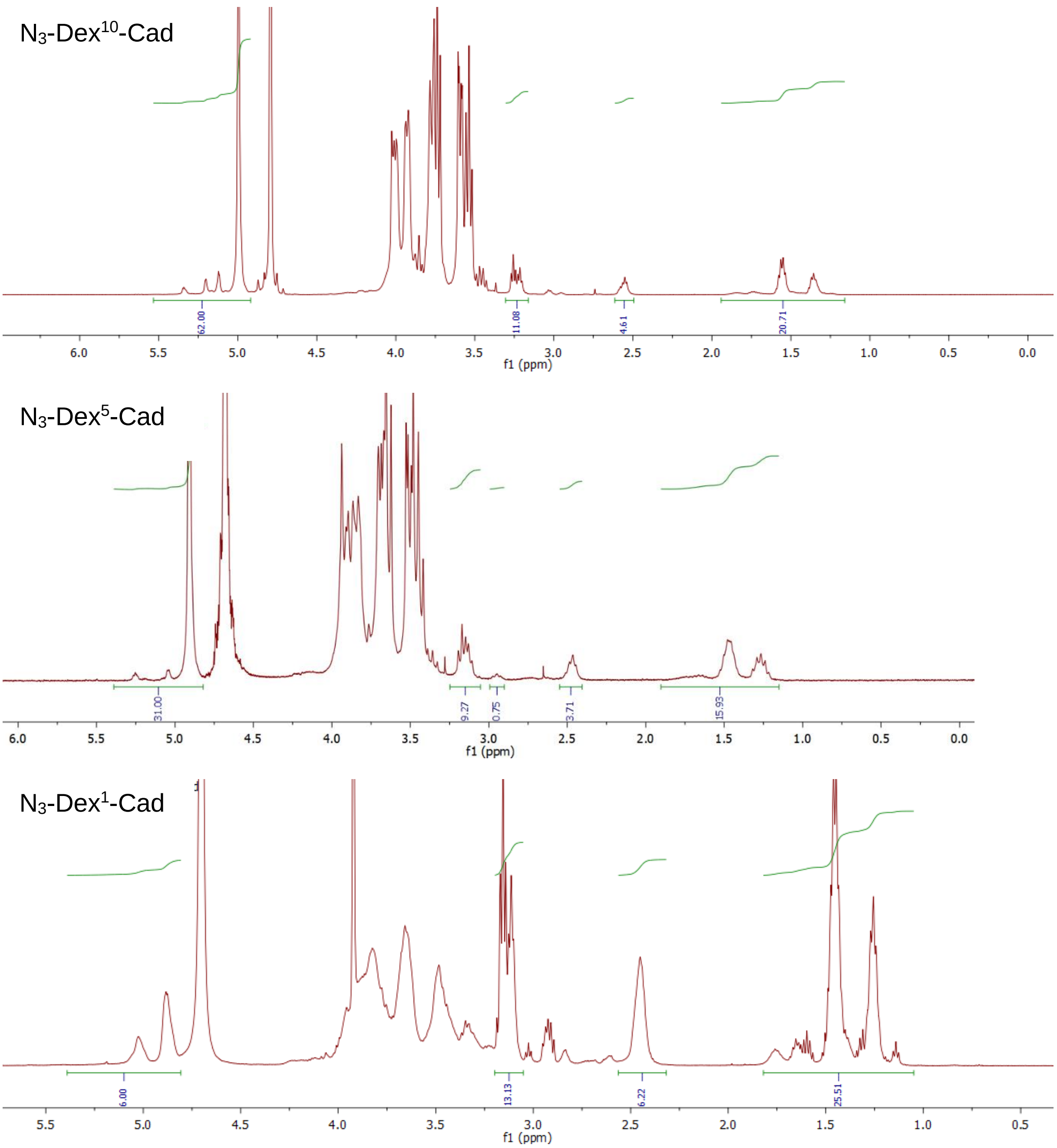

Figure S 5. ^{1}H-NMR spectra of N_3-Dex-Cad variants. ^{1}H-NMR (300 MHz, D_2O) δ = 6.24 – 4.80 (m, 1 H, C(1)H), 4.23 – 3.25 (m, C(2-6)H (glucose units); CH_2CH_2COOH; CH_2-N_3), 3.23 – 3.07 (m, NHCO-CH_2-N_3; CH_2-$(CH_2)_4$-NHCO-CH_2-N_3), 3.07 – 2.96 (m, 4 H, CH_2NH-BOC, $CH_2(CH_2)_4$NH-Boc), 2.73 – 2.34 (m, C**H**$_2$COOH), 1.80 – 1.19 (overlapped m, 15 H, C**H**$_2$$(CH_2)_3$NH-Boc, C**H**$_2$$(CH_2)_2$NH-Boc, C**H**$_2$$(CH_2)_1$NH-Boc, 3 C**H**$_3$(BOC); (1.80 – 1.37 C**H**$_2$-CH_2-NHCO-CH_2-N_3, C**H**$_2$-$(CH_2)_3$-NHCO-CH_2-N_3), 1.31 – 1.03 C**H**$_2$-$(CH_2)_2$-NHCO-CH_2-N_3)) ppm.

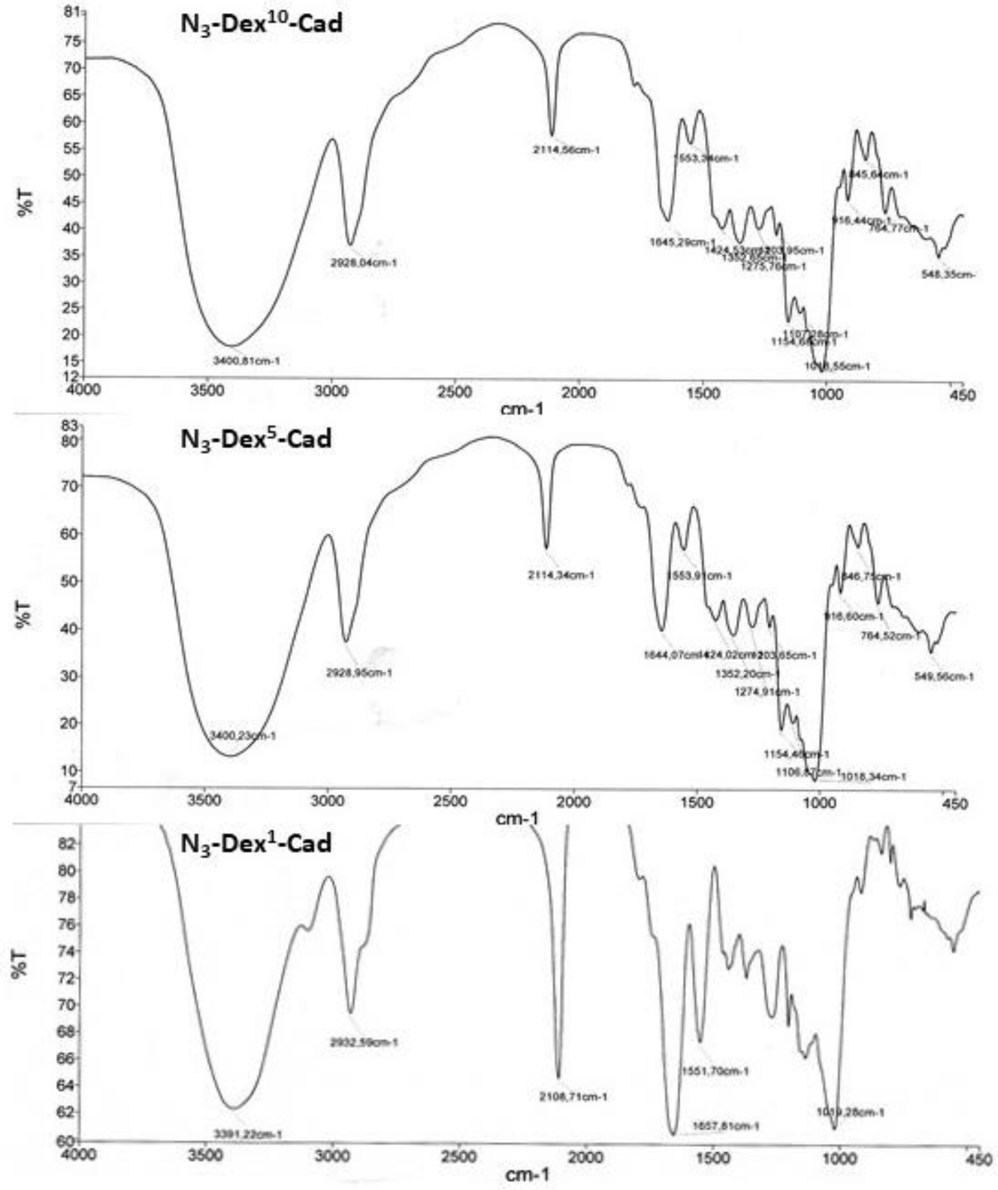

Figure S 6. IR-Spectra of N_3-Dex[10]-Cad variants. N_3-stretching vibrational band: 2110 – 2115 cm^{-1}[3].

1.5. Synthesis of BCN-IRDye700DX

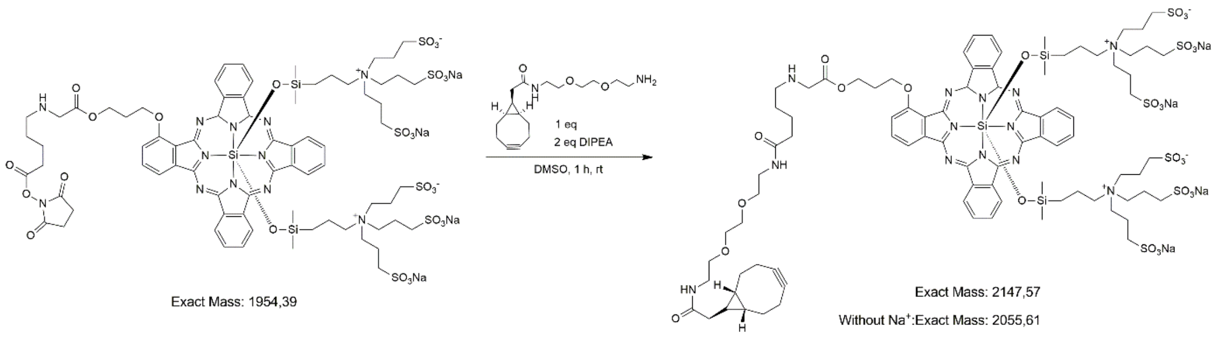

Scheme S 5 Synthesis of BCN-IRDye700DX.

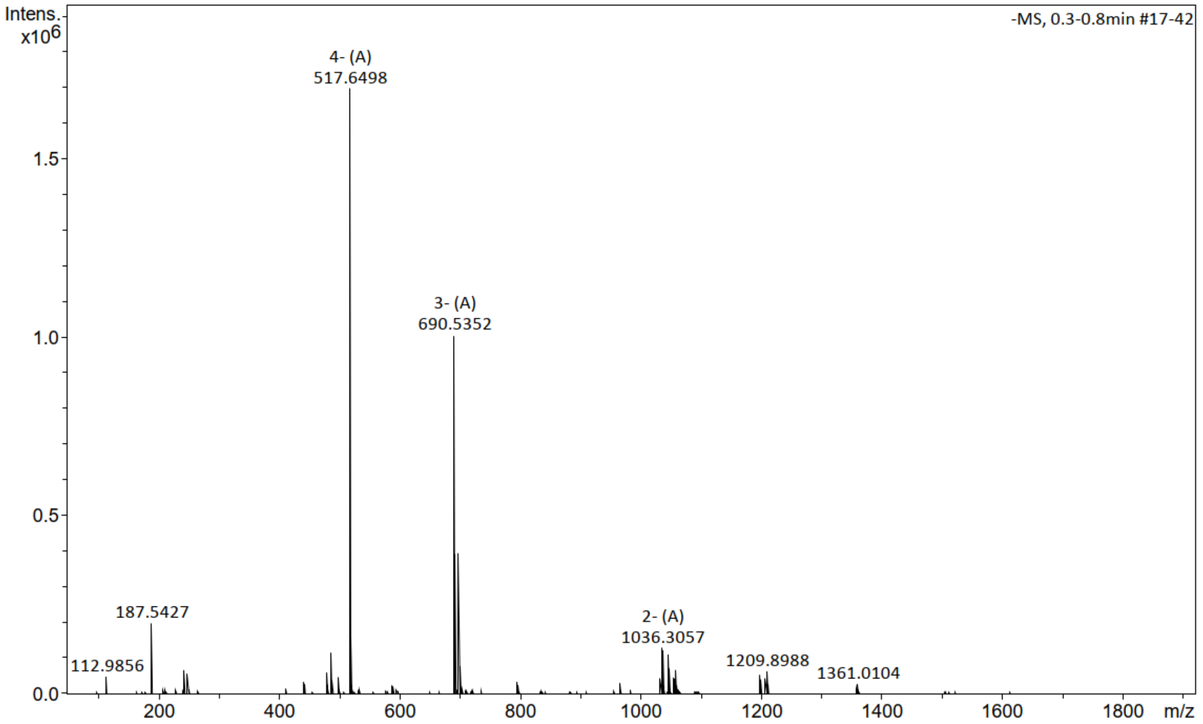

Figure S 7. ESI-MS spectrum of BCN-IRDye700DX®.

1.6. Structural formulas of utilized labels

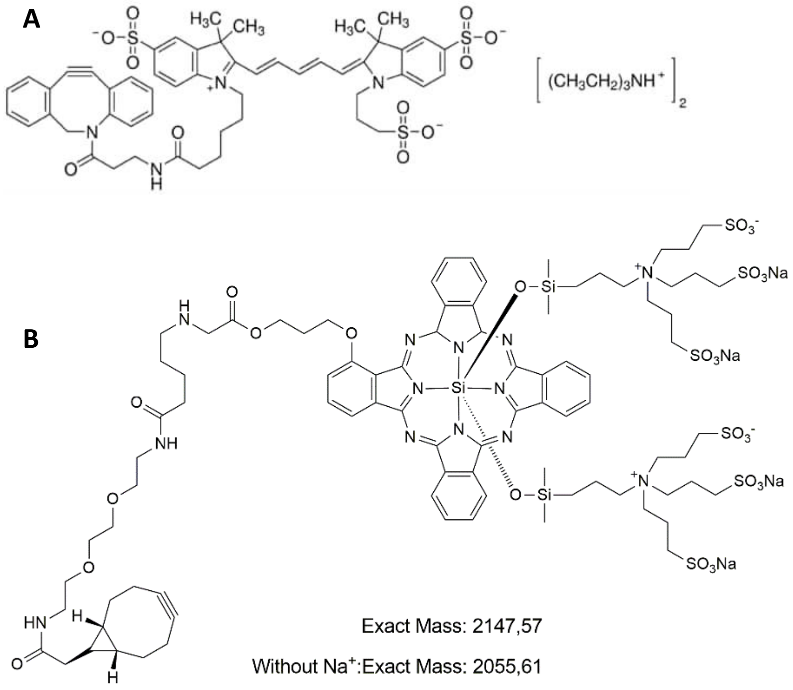

Scheme S 6. Utilized labels for SPAAC. **A**: DBCO-Cy5. **B**: BCN-IRsye700DX.

1.7. Generation of dextraknobs

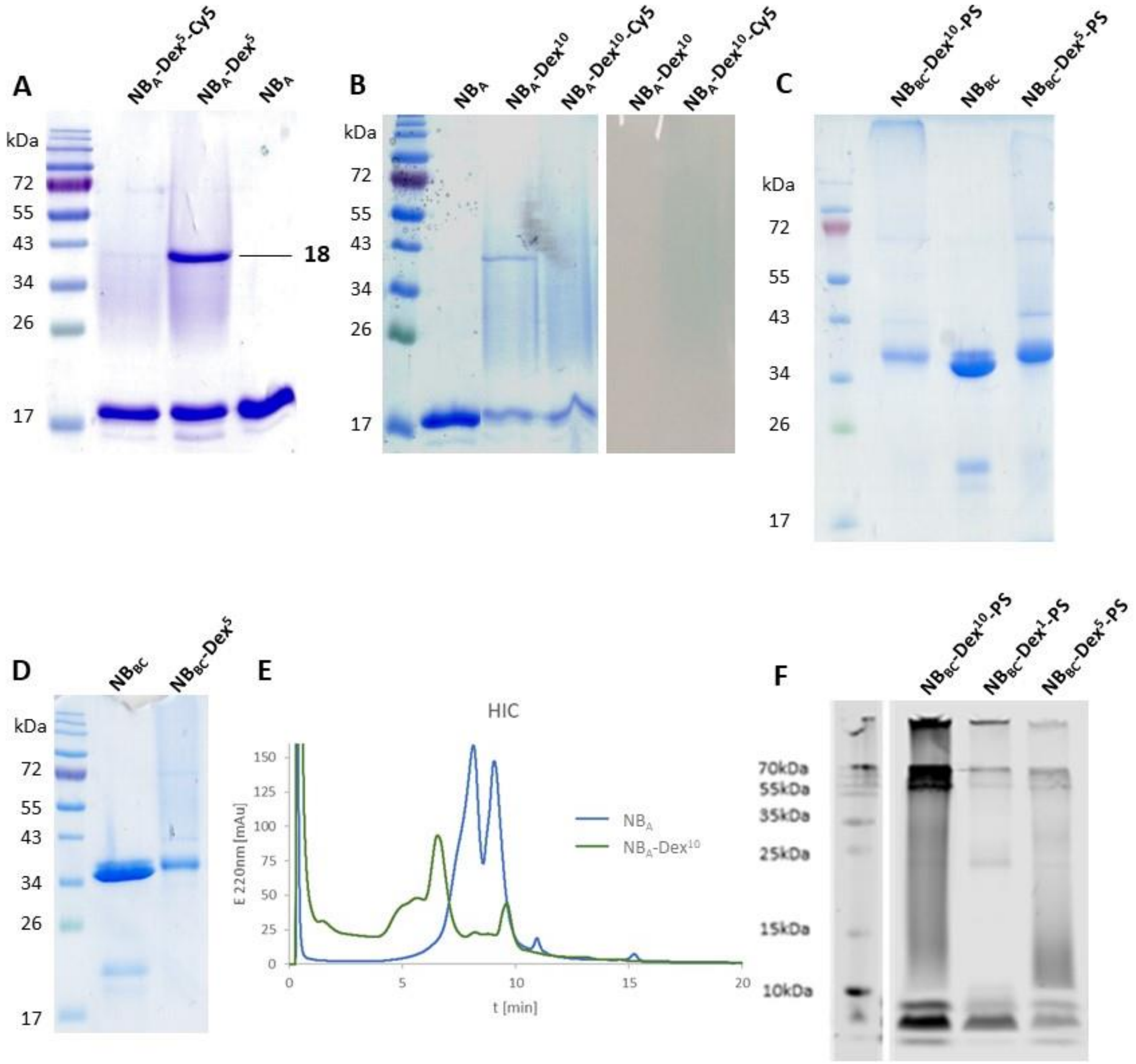

Figure S 8. A-D: Coomassie stained SDS-Gels of generated dextraknobs. **E**: HIC chromatogram of NBA-Dex[10]. **F**: SDS-Gel imaged with fluorescence reader (700 nm). Bands show excited IRDye700DX®.

2. Supplementary figures cell assays

2.1. Cellular binding assays

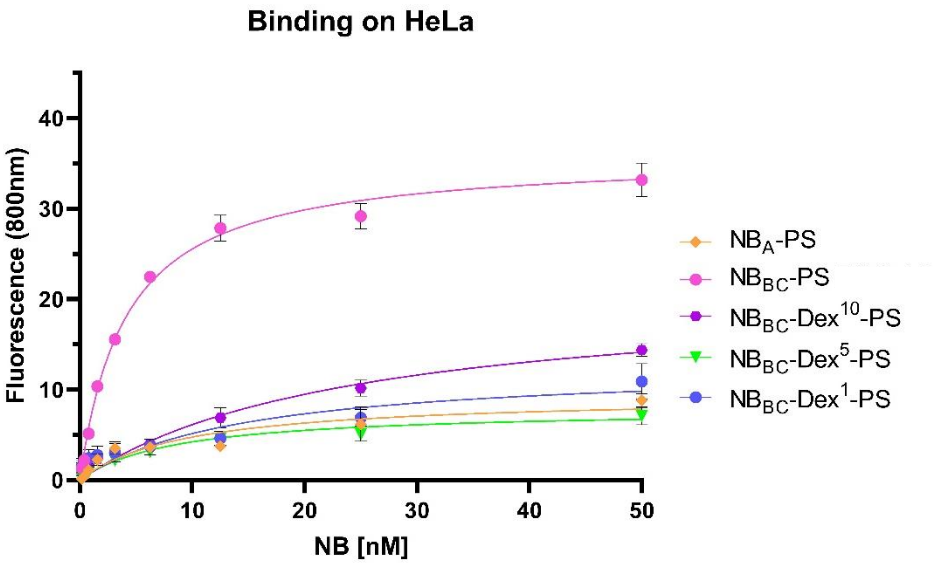

Figure S 9. Data of binding assay of NB-PS conjugates on HeLa cell line.

Table S 3. K_D-values of tested conjugates on HeLa cells.

	**K_D HeLa [nM]**
NB_{BC}-Dex^{10}-PS DOC 1.2	24.3
NB_{BC}-Dex^{5}-PS DOC 0.7	8.7
NB_{BC}-Dex^{1}-PS DOC 1.1	14.8
NB_{A}-PS-DOC 0.9	9.7
NB_{BC}-PS-DOC 1.5	4.1

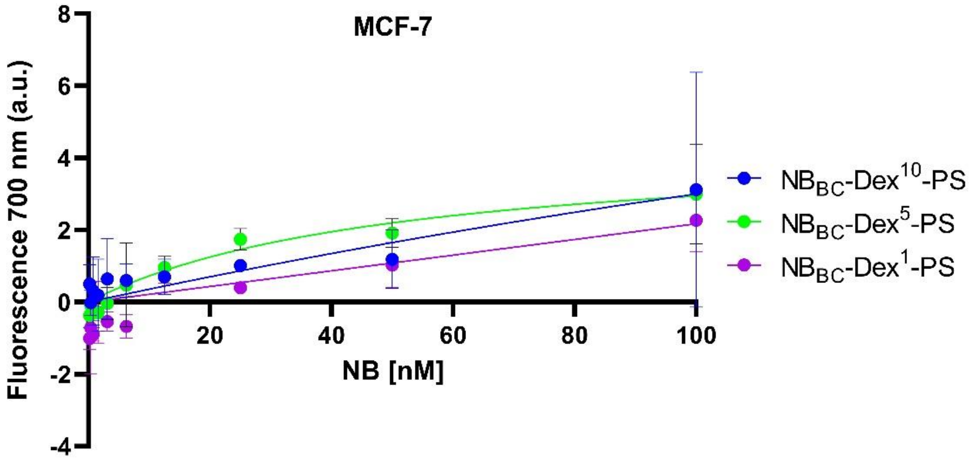

Figure S 10. Data of binding assay of NBBC-Dex-PS conjugates on MCF-7 cells.

2.2. Confocal microscopy of spheroids

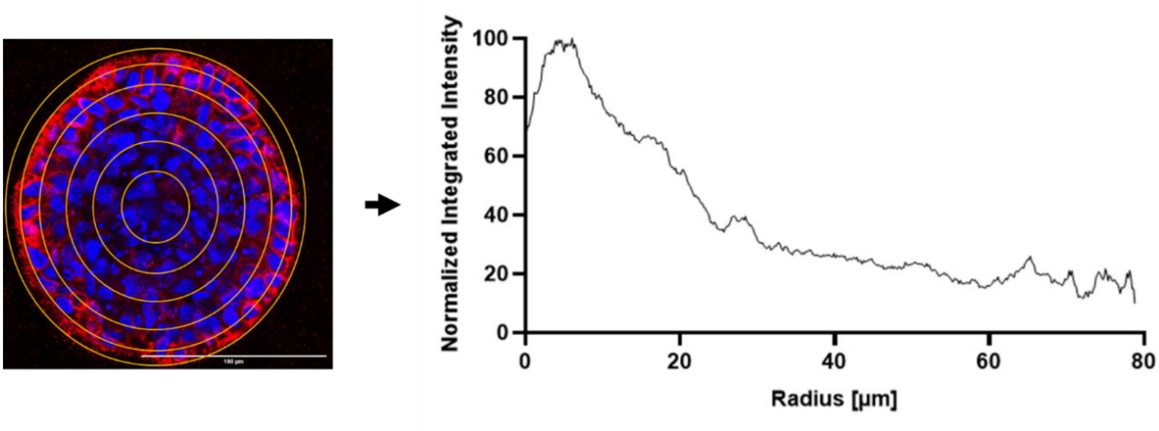

Figure S 11. Schematic representation of the analysis of confocal images taken of spheroids using ImageJ radial angle plug-in to create normalized integrated intensity plots of the NB signal along the radius of the spheroids.[7]

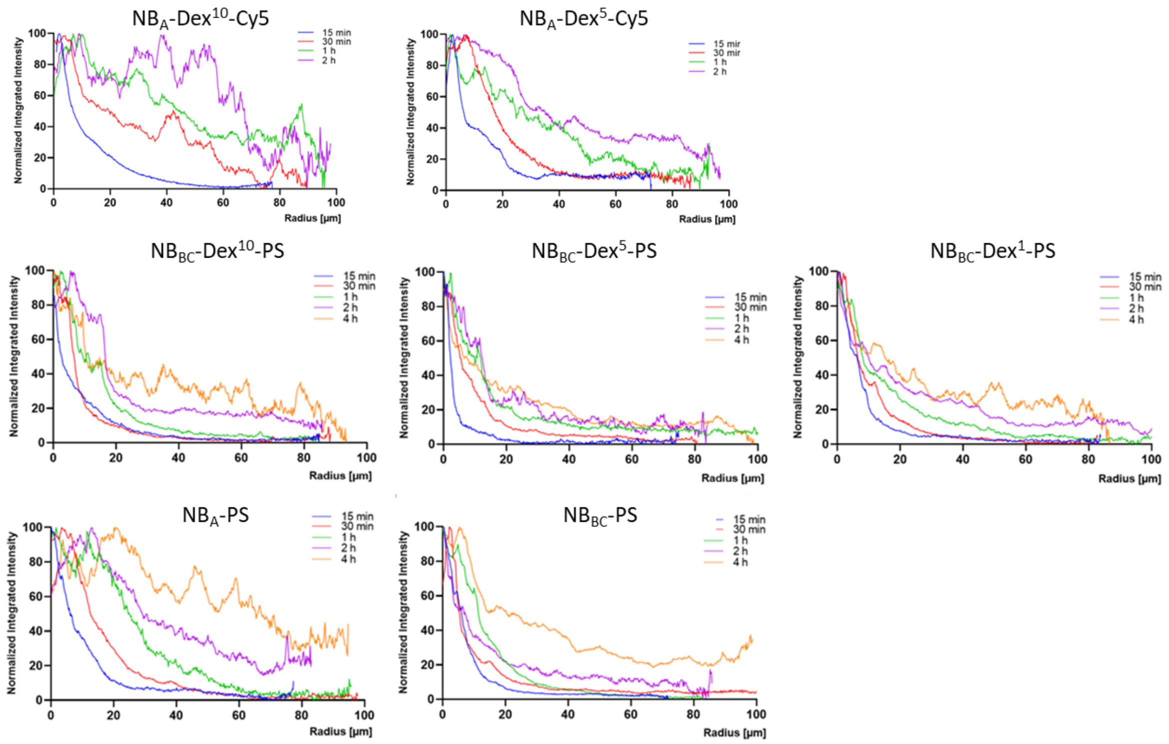

Figure S 12. Normalized integrated intensity plots of all conjugates at time points 15 min, 30 min, 1 h and 2 h. PS-conjugates were also measured at 4 h.

2.3. Nanobody-targeted photodynamic therapy (in vitro) on 2D monolayer cell culture

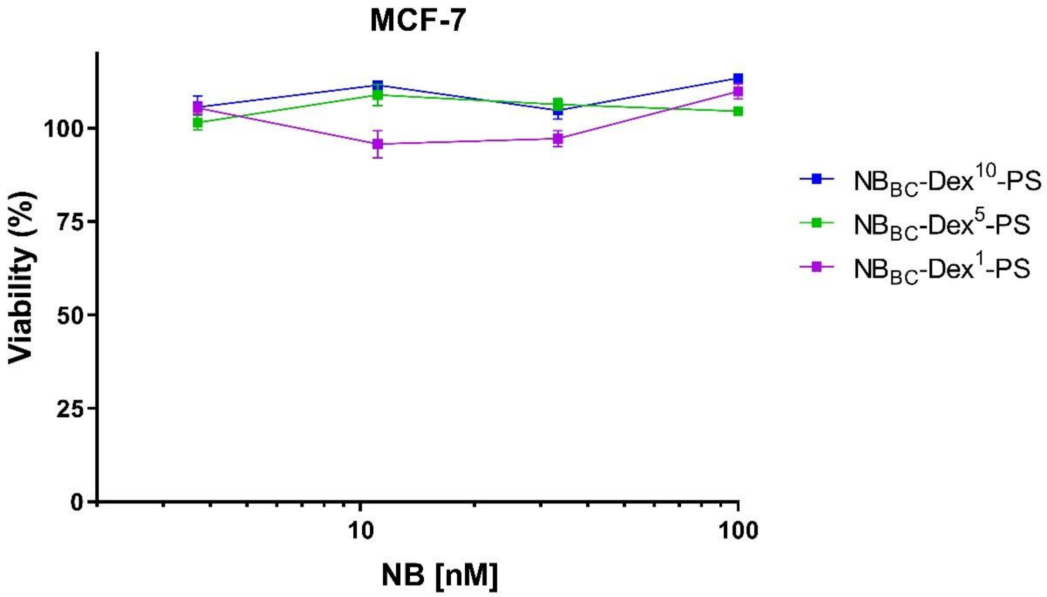

Figure S 13. Percentages (%) of cell viability after a 10 J/cm² light dose relative to untreated cells.

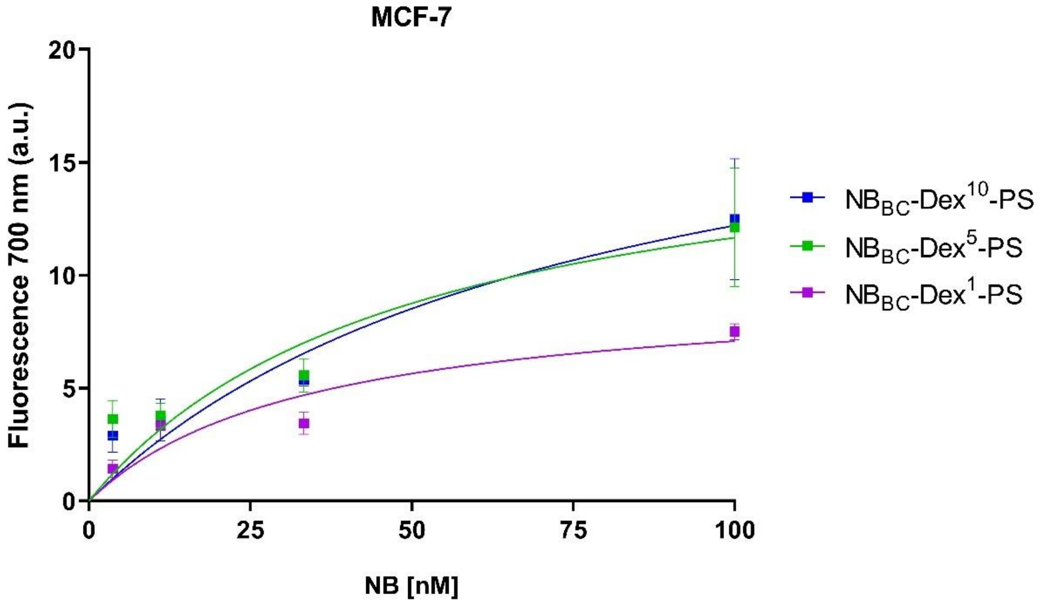

Figure S 14. Fluorescence intensities of NBBC-Dex--PS conjugates bound to cells after 30 min pulse incubation with a concentration range of the conjugates.

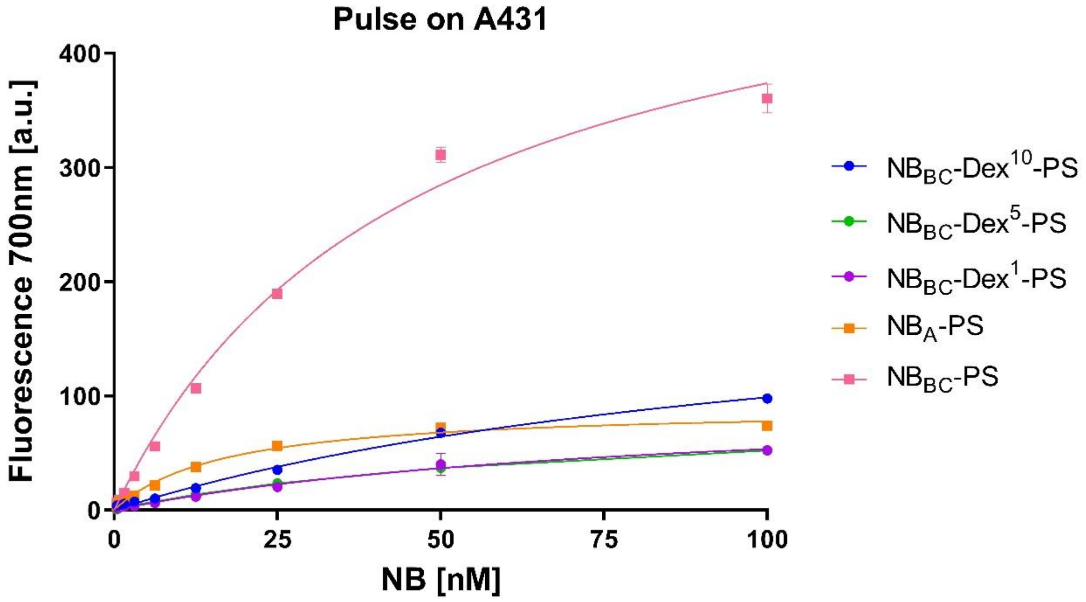

Figure S 15. Fluorescence intensities of NB-PS conjugates bound to cells after 30 min pulse incubation with a concentration range of the conjugates.

2.4. NB-targeted PDT on 3D spheroid cell culture

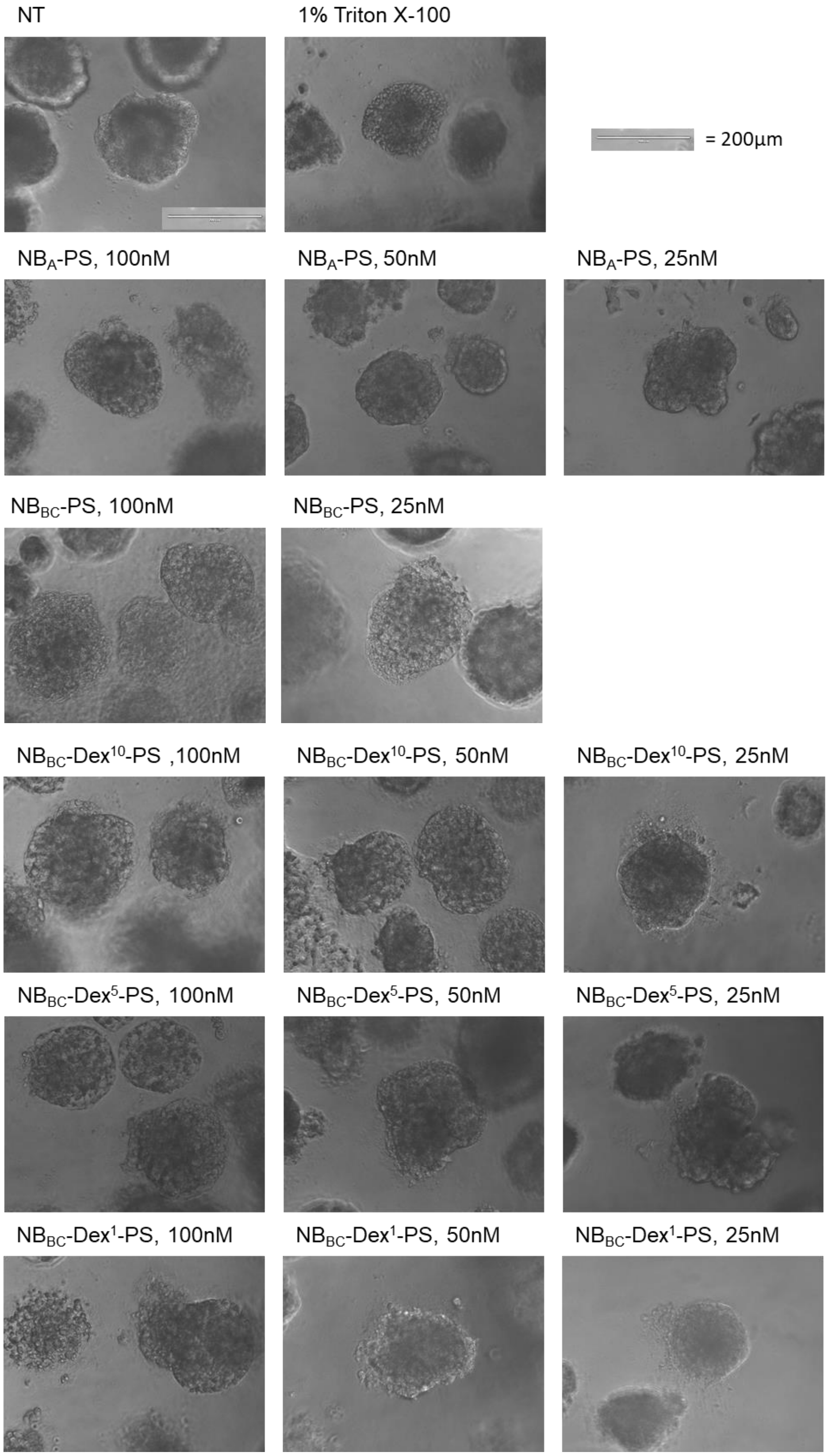

Figure S 16. 3D PDT 20J/cm², phase-contrast microscopy images taken 24h post treatment/irradiation.
